# A novel method for the multiplexed target enrichment of MinION next generation sequencing libraries using PCR-generated baits

**DOI:** 10.1093/nar/gkv773

**Published:** 2015-08-03

**Authors:** Timokratis Karamitros, Gkikas Magiorkinis

**Affiliations:** Department of Zoology, University of Oxford, Oxford OX1 3PS, UK

## Abstract

The enrichment of targeted regions within complex next generation sequencing libraries commonly uses biotinylated baits to capture the desired sequences. This method results in high read coverage over the targets and their flanking regions. Oxford Nanopore Technologies recently released an USB3.0-interfaced sequencer, the MinION. To date no particular method for enriching MinION libraries has been standardized. Here, using biotinylated PCR-generated baits in a novel approach, we describe a simple and efficient way for multiplexed enrichment of MinION libraries, overcoming technical limitations related with the chemistry of the sequencing-adapters and the length of the DNA fragments. Using *Phage Lambda* and *Escherichia coli* as models we selectively enrich for specific targets, significantly increasing the corresponding read-coverage, eliminating unwanted regions. We show that by capturing genomic fragments, which contain the target sequences, we recover reads extending targeted regions and thus can be used for the determination of potentially unknown flanking sequences. By pooling enriched libraries derived from two distinct *E. coli* strains and analyzing them in parallel, we demonstrate the efficiency of this method in multiplexed format. Crucially we evaluated the optimal bait size for large fragment libraries and we describe for the first time a standardized method for target enrichment in MinION platform.

## INTRODUCTION

The concept of using a nanopore as a biosensor was first proposed in the mid 1990s (1). More recently, the technical barriers that were preventing the generation of reliable DNA sequences were at least partially resolved (2–4), leading to the announcement of Oxford Nanopore Technologies’ MinION sequencer. This USB3.0-interfaced, portable device, which has been released in the frame of an early access program, is capable of producing more than 400 megabases of data in a 48-h run and delivering reads that can exceed 100 000 bp in length. However, the relatively low accuracy of the reads, which currently does not exceed 72% for the 2D (those computed from both strands) reads (5), demands increased read coverage for the generation of a reliable consensus sequence. Thus, the applications of this innovative sequencer are restricted to small genome studies, or to sequencing of long polymerase chain reaction (PCR) products. To expand these applications, we have developed a target enrichment method, specially adapted for MiNION nanopore sequencing libraries.

Methods that enrich NGS libraries for particular DNA sequences play pivotal role in the efficient sequencing of targets of interest, setting aside irrelevant regions (6,7). These methods rely on the hybridization of target sequences to molecular ‘baits’, which are actually single stranded oligos, most of the times conjugated with biotin (8,9). After hybridization, non-specific sequences are being washed away, and the following amplification of the captured library, using primers targeting the ligated sequencing-adapters, provides high read coverage around the targeted region.

The library preparation for MinION sequencer has some common parts with protocols used in other platforms, including the shearing of the DNA, the repair and the dA-tailing of the fragments’ ends. The ligation of Y-form and hairpin-form sequencing-adapters is platform-specific. The Y-form adapter is being ligated to the one end of the dsDNA fragments, allowing the attachment of the first stand to the sequencing nanopore, while the hairpin-like adapter is being ligated to the other end of the dsDNA fragments, allowing for the sequencing of both strands in series. Both the Y- and the hairpin- adapters are conjugated with special proteins (Motor enzyme, HP motor, Tether) that allow control of the speed during the translocation of the strands through the nanopores and promote saturation of the fragments of the basement membrane of the sensor chip (Supplementary Figure S1). These proteins pose restrictions for downstream processing of the library, for example the breaking of the biotin-streptavidine bond, which is needed during in-house developed protocols for target enrichment purposes (10).

Crucially, the very special form of the resulting DNA fragments of MinION libraries and their remarkable length, restrict their compatibility with standard ready-to-use target enrichment kits, like IDT xGen, Agilent SureSelect etc. These kits have been standardized for use with specific platforms (Illumina, IonTorrent etc.) and they use small biotinylated probes to capture single stranded DNA molecules (usually <1000 bp), which are post-capture released with PCR, using primers against the initially ligated sequencing-adapters. MinION sequencing-adapters cannot withstand the PCR thermal conditions due to their increased sensitivity and special shape. On the other hand, the potential use of these commercial kits without any initial sequence modification would result in the release of single-stranded molecules, which cannot be ligated with the double stranded MinION adapters.

In this study, we overcome the addressed limitations by capturing the targeted sequences before the ligation of these sensitive sequencing-adapters. Briefly, we utilize the Y-shaped PCR-adapters (note these are different from the Y-shaped sequencing-adapters) and their compatible primers that are used for the amplification of low input genomic DNA libraries and are contained in the ‘MAP003 genomic sequencing kit’ (Adapter Short Y, Table 1). To date, these adapters have not been standardized for use with the commercially available kits. Performing the ligation of these adapters on 1ug of sheared genomic DNA, we were able to capture the target sequences and then amplify-release them from the baits, using compatible primers. Also, by the addition of specific barcode sequences to these primers we simultaneously tagged the libraries thus allowing for pooling of multiple libraries in a single run. We then finalized the library preparation based on MinION nanopore's protocol, and loaded the sequencer for a 48 h genomic DNA sequencing run. Using Lambda Phage, *E. coli* O157:H7 (str. Sakai) and *E. coli* W3110 (str. K12) DNA as models, we describe a method that effectively enriches specific targets and, crucially, their flanking sequences. This particular feature, in combination with the very unique characteristic of MinION to produce extremely long reads can be very useful for structural studies, such as the identification of viral integration sites in large genomes. The limited capacity (∼400 Mbases) of a single MinION flowcell is not adequate to cover the human genome in a WGS experiment. Thus, the described method could be used for targeting known sequences of viral or retroviral elements and capturing long flanking fragments that would allow mapping their integration site.

**Table 1.** Adapters and primers. Oligos in *italics* have been described in the MAP community at: *https://wiki.nanoporetech.com/display/BP/Adapter+sequences*

Oligo Name	Sequence (5’-3’)
*AdapterY_SK23*	30(C3 spacer)-12(T)-4(C18 spacer)-GGTTGTTTCTGTTGGTGCTGATATTGCGGCGTCTGCTTGGGTGTTTAACCT
*AdapterY_SK24*	GGTTAAACACCCAAGCAGACGCCGCAATATCAGCACCAACAGAAACAACCTTTGAGGCGAGCGGTCAA
*Hairpin_SK26*	Phosphate-CGTTCTGTTTATGTTTCTTGGACACTGATTGACACGGTTTAGTAGAAC-4(C3 spacer)-28(T)-CAAGAAACATAAACAGAACGT
*AdapterY_Short_LI32*	GGTTGTTTCTGTTGGTGCTGATATTGCGGCGTCTGCTTGGGTGTTTAACCT
*AdapterY_Short_LI33*	Phosphate-GGTTAAACACCCAAGCAGACGCCGAAGATAGAGCGACAGGCAAGTTTTGAGGCGAGCGGTCAA
*PCR_primer_PR2*	Phosphate-TTTCTGTTGGTGCTGATATTGC
*PCR_primer_3580F*	Phosphate-ACTTGCCTGTCGCTCTATCTTC
*Bar01_PR2_F*	GGTGCTGAAGAAAGTTGTCGGTGTCTTTGTGTTAACCTTTTCTGTTGGTGCTGATATTGC
*Bar02_PR2_F*	GGTGCTGTCGATTCCGTTTGTAGTCGTCTGTTTAACCTATTTCTGTTGGTGCTGATATTGC
Bio-T (10)	Biotin-TCAAGGACATCCG
B (10)	CGGATGTCCTTG
L1F	GGATTTCCGTCGGGCAGTAT
L1R	CACACTCTGGCTGATGGACG
L3F	GGAAAGCGAGATGGGGAGAC
L3MR	CATTTGGCTGTCCAAGCTCC
L3MF	TATCAACCCGGAGCTTGGAC
L3R	TAGCCGCTTCGGTTCATCAG
L4F	GATGAACCGAAGCGGCTAAAG
L4MR	CCGTCACGCACATGGGAT
L4MF	GGATCCCATGTGCGTGAC
L4R	CATCATGCAGCTTCCCTCCC
L5F	TGGAGGGCAGCTTGATTTCG
L5MR	CTGGTGCGTTTCGTTGGAAG
L5MF	ATACCTTCCAACGAAACGCAC
L5R	ACACACGTGAACTTCCAGCA
EC1F	AAATTGAAGAGTTTGATCATGGCTC
EC1R	GACTTAACAAACCGCCTGCGT
EC2F	CGCAGGCGGTTTGTTAAGTC
EC2R	TCCAGTTTATCACTGGCAGTCT
EC3F	GCCAGTGATAAACTGGAGG
EC3R	CAGGCGCTCTCCCAG
EC4F	CTGCTTTGCACGCAGGAG
EC4R	CGAGCTCACAGTATGTGCATTTT
EC5F	TACTGTGAGCTCGATGAGTAG
EC5R	GCCGTATGTCTCCCGT
EC6F	ACGGGAGACATACGGCGG
EC6R	GCCTCGTCATCACGCCTC
EC7F	GCGTGATGACGAGGCACT
EC7R	CACATCAAGGCTCAATGTTCA
EC8F	CATTGAGCCTTGATGTGTAGGA
EC8R	CGAACACCAGTGATGCGTCC
EC9F	GCATCACTGGTGTTCGGGT
EC9R	AGCGATAACTCGAGGCTTCTTA

Finally, we demonstrate the ability of this method to enrich and simultaneously tag more than one MinION sequencing libraries to be analyzed in the same run, in a multiplexed format.

## MATERIALS AND METHODS

### Baits’ length and genomic DNA shearing optimization

We generated the baits used in this study through multiple PCR reactions. For the optimization of the method, we aimed to design baits of three different lengths (250 bp, 450 bp, 900 bp). Twelve primers (Sigma Aldrich, Gillingham, UK) (primers L3F to L5R in Table 1), in combination with Platinum Taq DNA Polymerase (10966–018, Invitrogen, Carlsbad, CA) produced 6 baits of average length 242 bp (short-‘S’), or 3 baits of average length 460 bp (medium-‘M’) or 2 baits of average length 884 bp (long-‘L’) (Supplementary Figure S2). They were all targeting a 1317 bp region of *Phage Lambda* genome (accession number NC_001416.1, nt. 41 053 – 42 370). Based on the hybridization method described below, we combined these 3 bait-pools with *Phage Lambda* DNA, after shearing it at approximately 5000 bp (‘5K’) and 10 000 bp (‘10K’) fragments with g-Tubes (520079, Covaris, Woburn, Massachusetts, USA), creating six combinations in total: S-5K, S-10K, M-5K, M-10K, L-5K, L-10K.

### Assessment of the enrichment

We assessed the capture of the target sequences for each individual preparation with qPCR before the ligation of the sequencing adapters. We used ‘Power SYBR® Green PCR Master Mix’ (4368577, Invitrogen, Carlsbad, CA) (thermal conditions: 50°C for 2 min, 95°C for 2 min and 40 cycles of 95°C for 20 s / 60°C for 1 min) with primers L1F-L1R for amplifying the untargeted regions, and primers L4MF-L5MR for amplifying the targeted regions of Lambda genome (Table 1, Supplementary Figure S2). We constructed standards by serial dilutions of *Phage Lambda* genome ranging from 10^6^ to 10^0^ copies/ul and we calculated the absolute copies of each PCR product per ul of enriched library or genomic control DNA (Supplementary Figure S3). Finally we calculated the ratio of targeted to untargeted copies/ul for each library preparation and for the pre-captured genomic control DNA (Figure 2).

### Target enrichment of *Phage Lambda* and *E. coli* loci

We used the ‘M’ pool of baits for the enrichment of the 1317 bp-long target of *Phage Lambda* genome. For the enrichment of the rRNA operons of *E. coli* strains O157:H7 (accession number NC_002695.1) and K12/W3110 (accession number NC_007779.1) we generated 9 baits of average length 597 bp (primers EC1F to EC9R in Table 1) targeting the 5258 bp-long rrnH operon of strain O157:H7 (nt. 227102–232360), which contains genes for the 16S (rrsH), 23S (rrlH) and 5S (rrfH) rRNA, but also the genes for alanine (alaV), isoleucine (ileV) and aspartate (aspU) tRNAs.

We gel-extracted the PCR products with QIAquick Gel Extraction Kit (28704, Qiagen, Hilden, DE) and we assessed their concentration using the Quant-iT PicoGreen dsDNA kit (P11496, Invitrogen, Carlsbad, CA). We mixed them in equimolar concentrations, blunt end-repaired them using NEBNext End-Repair module (E6050S, New England BioLabs, Hitchin, UK) and purified them with Agencourt AMPure XP PCR Purification beads (A63880, Beckman Coulter, High Wycombe, UK). We rehydrated the biotin conjugated oligos (10) (Table 1) (Sigma Aldrich, Gillingham, UK) directly with hybridization buffer (10mM Tris 1mM EDTA pH 7.5–8.0, 50 mM NaCl) and gradually annealed them to form blunt-ended biotin adapters as described in (10). We ligated them with the mixed PCR products using NEBNext Blunt/TA Ligase (M0367S, New England BioLabs, Hitchin, UK) and we purified the produced baits with AMPure XP beads.

In parallel, we sheared 1 ug of genomic DNA to approximately 5000 bp. We end-repaired the sheared DNA with NEBNext End Repair module, purified it with AMPure XP beads and dA-tailed it with NEBNext dA-tailing module (E6053S, New England BioLabs, Hitchin, UK). Finally we ligated the 3’end T-overhanging Y-formed PCR-adapters that were included in the MAP003-MinION gDNA Sequencing Kit (Adapter Short_Y_LI32—LI33, Table 1) to the d-A tailed DNA.

The hybridization of the pooled biotin-baits with the PCR-adapter-ligated DNA was based on the protocol of SeqCap Hybridization and Wash Kit (05634261001, Roche Diagnostics, Indianapolis, IN, USA), after the modification of the initial hybridization mixture. We mixed 500ng of PCR-adapter-ligated Lambda DNA (15ul), 2x Hybridization buffer (final volume/2 ul) and Hybridization component A (final volume/10 ul). After incubating the mixture at 95°C for 5min, we added 8ul of baits-mix (diluted in TE, to the ratio: baits copies / target copies = 1000). The hybrids were captured on the streptavidin-coated Dynabeads M-270 (65305, Invitrogen, Carlsbad, CA) while off-target fragments were removed during the following washing steps. We amplified the captured *Phage Lambda* library using the MinION provided primers (PCR primer PR2—3580F, Table 1) targeting the ligated PCR-adapters and the LongAmp Taq 2x Master Mix (M0287S, New England BioLabs, Hitchin, UK). We added barcoding sequences at the forward primer used in the amplification of *E. coli* strains O157:H7 and K12/W3110 enriched libraries (PCR primer Bar01_PR2_F, Bar02_PR2_F, Table 1). In a post-PCR magnetic separation, we removed the beads and the baits and we purified the remaining PCR product using AMPure XP beads, optimized to select DNA fragments larger that 1000 bp. *E. coli* O157:H7 and K12/W3110 libraries were pooled and then analyzed in the same MinION run.

**Figure 1.**
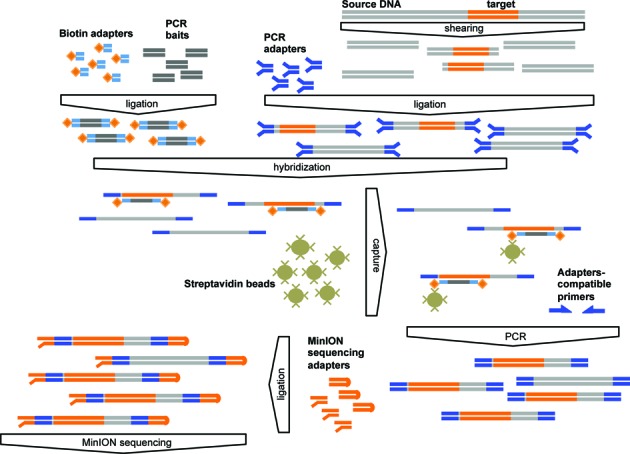
Schematic representation of the target enrichment process. Biotin-adapters (orange rhombus and blue bars) ligated on PCR-generated baits (dark gray bars) are hybridized with the target sequences (orange bars), which are weakly represented within the PCR-adapter-ligated (light blue) sheared DNA. The capture on the streptavidin beads and the magnetic separation of the hybrids enriches for the target sequences. PCR-adapter-compatible primers (blue arrows) amplify-release the fragments from the beads before the ligation of the sequencing-adapters (orange hairpin- and Y-shaped) and the loading to the MinION flowcell.

We completed the libraries preparation with a second round of end-repair, dA-tailing and sequencing-adapters ligation (Adapter Y_SK23 – SK24, Table 1) according to MinION nanopore's protocol, after adding the provided internal control (CS-DNA) corresponding to the 3’-end of Lambda genome. Finally, we conditioned and loaded the libraries to the sequencer for a 48 h run, reloading the sequencer every 12 h. The overall procedure is summarized in Figure 1.

### Bioinformatics and statistical analysis

We performed the base calling via the Metrichor agent (provided by Oxford Nanopore Technologies). We converted the *.fast5* reads to *.fasta* files using the poRe package for R programming language (Bioinformatics, 2014, doi: 10.1093/bioinformatics/btu590). PoRe package was also used to assess the read-length characteristics of the sequencer for each enrichment experiment. For the *E. coli* libraries, we filtered out the reads corresponding to the Lambda control DNA after mapping them against the genome of the phage. We separated the *E. coli* O157:H7 and K12/W3110 reads using the 38nt-long barcode sequences as queries in a BLAST analysis, after generating a BLAST database from the *.fasta* reads. We performed the mapping alignment of the reads using LAST (BMC Bioinformatics, 2010, doi: 10.1186/1471-2105-11-80) setting parameters -T = 1, -Q = 0, -a = 1, thus allowing only complete reads to be mapped. The resulting *.maf* alignments were converted to *.sam* using ‘maf-convert’ Python script. Samtools (Bioinformatics, 2009, doi: 10.1093/bioinformatics/btp352) were used to generated .bam files and alignment statistics. We visualized mapping alignments with IGV (Briefings in bioinformatics, 2013, doi: 10.1093/bib/bbs017).

We performed coverage analysis across the genomes tested using ‘bedtools’ (Bioinformatics, 2010, doi: 10.1093/bioinformatics/btq033). To calculate the mean coverage across targeted and untargeted regions, we segregated the reference *Phage Lambda* genome in overlapping-by-100 bp, 200 bp-sized windows and *E. coli* genomes in overlapping-by-1000 bp, 2000 bp-sized windows, using ‘bedtools-makewindows’ and then for each individual window we calculated the coverage using default settings of ‘bedtools-map’. We calculated the per-window normalized coverage after dividing the coverage of each window by the total mapped reads of each *.bam* file. We evaluated the performance of the method using ‘bedtools-coverage’ to count the mapped reads on the targeted and on the untargeted regions of *Phage Lambda* genome, before and after the enrichment. We also used ‘bedtools-coverage’ across all enrichment experiments to measure the sensitivity—the percentage of the target bases that are represented by one or more reads—and the specificity—the percentage of reads that map to the intended targets—of the method (6).

We estimated the error rate of the sequencer across the enrichment experiments using BLAST (mean percentage of output identity), after building databases for each individual 2D and single-stranded (template) pool of reads and setting -gapopen and the -gapextend parameters to 0 and 2, respectively.

## RESULTS

Using PCR-generated biotinylated probes and pre-enrichment ligated PCR-adapters, we targeted confined regions of *Phage Lambda* and *E. coli* genomic libraries. In a post-enrichment step we ligated the MinION sequencing-adapters and performed single and multiplexed 48 h sequencing runs.

### Assessment of the baits size and genomic DNA library size

**Figure 2.**
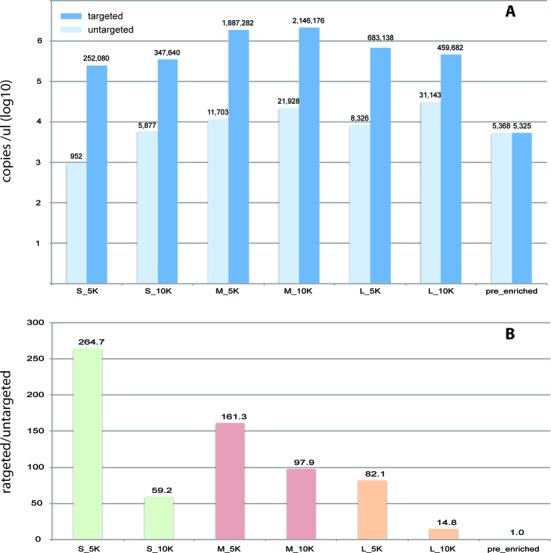
Quantitative assessment of the enrichment process with qPCR. (**A**) Absolute concentration (copies/ul) of targeted (dark blue) and untargeted (light blue) regions of *Phage Lambda* genome with respect to the combination of the baits (‘S’-short, ‘M’-medium, ‘L’-long) and the DNA shearing size (5 Kbp or 10 Kbp) used during the enrichment. The same regions were also amplified using control (pre-enriched) DNA as template. (**B**) Ratio of the concentration of the targeted regions versus the concentration of the untargeted regions.

In order to find the best combination of baits length and genomic DNA fragmentation size we compared the copies of the targeted versus the untargeted regions of the *Phage Lambda* genome, both in pre-enriched control DNA and in 6 enriched libraries: S-5K, S-10K, M-5K, M-10K, L-5K, L-10K (see details in Materials and Methods). Before the enrichment process, no significant difference in the concentration of the targeted versus the untargeted regions was observed as they were equally represented in the starting material. The ratio of the absolute concentration of the targeted sequences (per ul of enriched library) versus the concentration of the untargeted sequences was higher in the S-5K (264.7) and the M-5K (161.3) libraries, while the total yield of targeted copies was approximately 1 log increased in the M-5K library compared to the S-5K (1.8×10^6^ versus 2.5×10^5^). The enrichment performance was constantly decreased using the 10K fragmentation size independently from the baits’ length (Figure 2, Supplementary Figure S3).

### Pre- and post-capture read coverage across *Phage Lambda* genome

**Figure 3.**
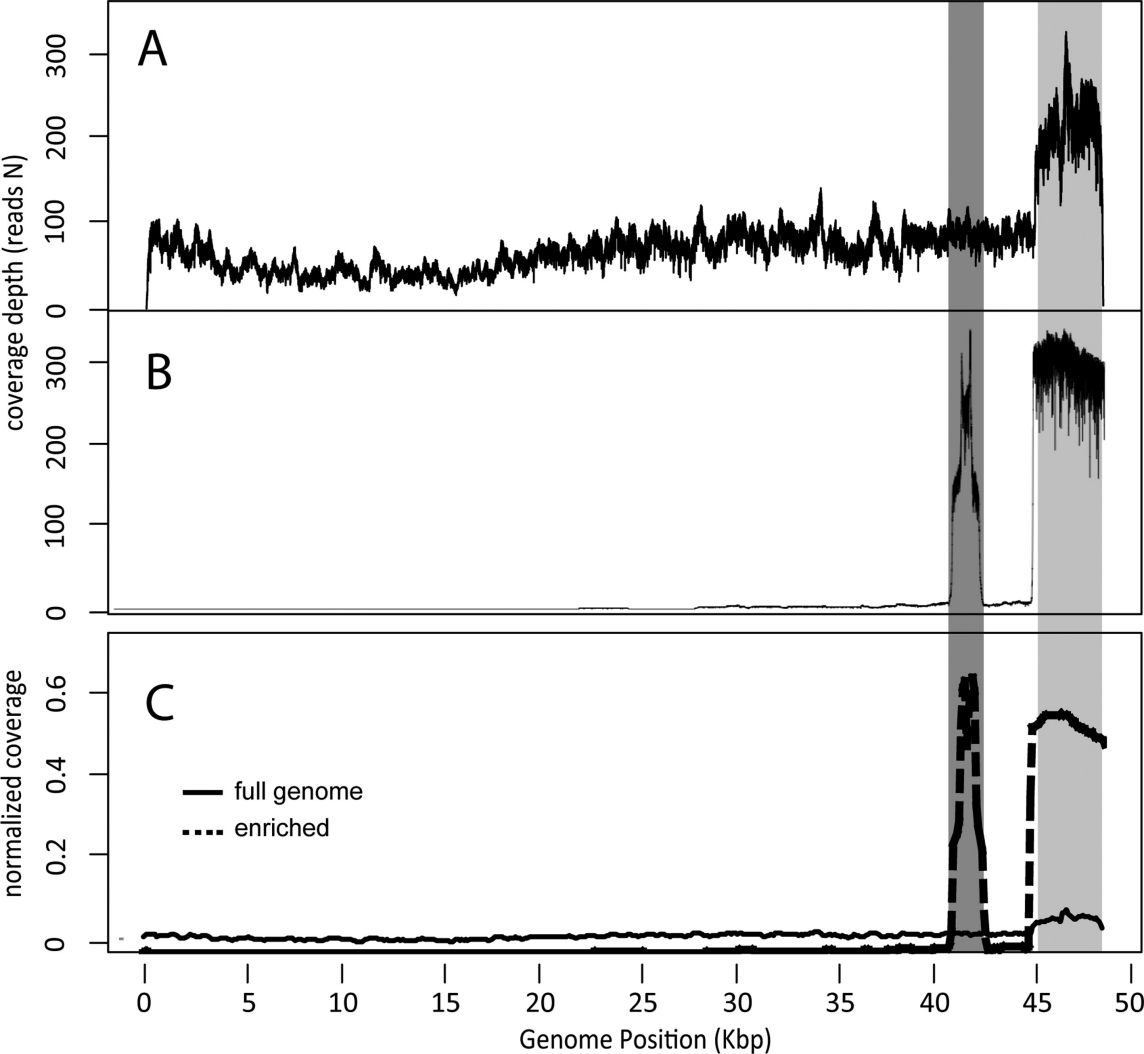
(**A**) Raw (per-base) coverage in whole genome sequencing of *Phage Lambda*. (**B**) Raw (per-base) coverage after enriching the sequencing library for a specific region (41 053 – 42 351 bp, dark gray column). (**C**) Normalized coverage across the genome (segregated in overlapping-by-100 bp 200 bp-sized windows). Enriched and whole genome library coverage is plotted with dashed and solid line, respectively. The higher coverage across the last 5 kb of the genome (light gray column) corresponds to the internal control DNA added to both libraries.

For comparison purposes, we demonstrate a control whole genome sequencing experiment of the *Phage Lambda* DNA. The raw (per base) and normalized coverage in the whole-genome and in the target-enrichment sequencing experiment are shown in Figure 3. The read coverage at the 3’ end of the genome appears to be increased compared to the rest of the genome, as this region corresponds to the internal control DNA added at the beginning of the library preparation. This region has been excluded from any further comparative analysis.

The percentage of the reads mapped on the targeted region in the enrichment experiment was significantly increased compared to those mapped on the same region in the whole genome sequencing experiment (95.7% versus 18.4%, Fishers’ exact test *P* < 0.0001) (Table 2).

**Table 2.** Evaluation of *Phage Lambda* target enrichment process. Sequencing reads distribution

	Reads on targeted region*	Reads on untargeted regions**	Total reads mapped on genome
	N (%)	N (%)	N (%)
Whole genome sequencing	247 (18.4)	1092 (81.6)	1339 (100.0)
Target enrichment	1179 (95.7)	53 (4.3)	1232 (100.0)

*Phage Lambda target: 41 053 – 42 370 bp.

**Untargeted region corresponding to the control DNA has been excluded.

### Capture of reads at target-flanking regions

**Figure 4.**
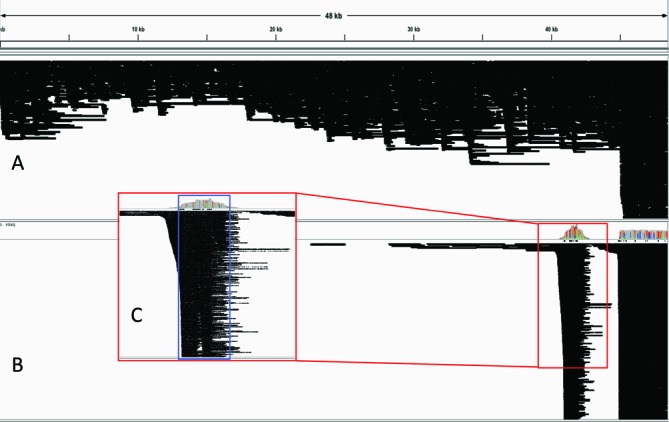
IGV screenshots from (**A**) mapping alignment of reads generated from the full genome sequencing of *Phage Lambda* and (**B**) mapping alignment of reads generated after the enrichment for the targeted region. (**C**) Magnified view of the targeted region (blue box) with visible captured long reads exceeding the target coordinates for over 1000 bp.

The read coverage was also increased at the regions spanning the targeted sequences. This was due to the capture of long DNA fragments, which were partially mapped on the target, spanning the target coordinates (Figure 4). We could accurately remap randomly selected off-target portions of high-quality reads on the same (off-target) *Phage Lambda* coordinates, using BLAST (Supplementary Figure S4).

### Multiplexed target enrichment of *E. coli* rRNA operons

Each bacterial genome tested contains seven highly conserved rRNA operons. The PCR-generated baits were targeting the rrnH operon of *E. coli* O157:H7, which contains the tRNA genes alaV, ileV and aspU.

**Figure 5.**
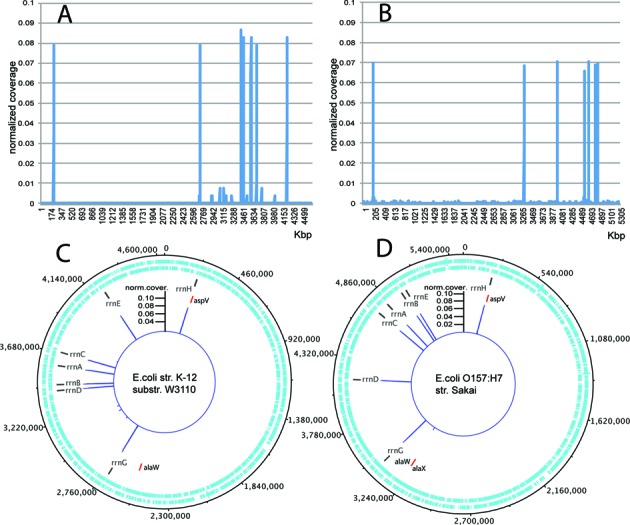
(**A** and **B**) Normalized coverage across overlapping-by-1000 bp, 2000 bp-sized genome windows of *E. coli* O157:H7 and K12/W3110, respectively. (**C** and **D**) Normalized coverage across genome maps of the same strains, indicating the positions of rRNA operons (black bars) and the positions of off-operon tRNAs (red bars) that are potential targets of the enrichment.

All operons within the two genomes were captured, according to the coverage analysis over the target regions. Off-operon tRNA genes, was also expected to be captured due to their similarity to rrnH operon-integrated tRNA genes. Off-operon tRNA genes aspV, alaW and 5S solo rRNA gene rrfF (119 bp) were not captured during the enrichment of *E. coli* K12/W3110. In the case of *E. coli* O157:H7, off-operon tRNA genes aspV, alaX and alaW were successfully captured, despite their small size (75 bp each) (Figure 5, Table 3).

**Table 3.** Coverage and total reads mapped over the targets. Sensitivity of the method

	Target locus	Target coordinates (bp)	Reads on target (N)	Target length (bp)	Target bases covered (Sensitivity -%)
*E. coli* O157:H7	rrnH	227 102 – 232 204	330	5102	5102 (100.0)
str. Sakai	aspV	240 481 – 240 557	4	76	76 (100.0)
accession:	alaX	3 244 824 – 3 244 899	2	75	75 (100.0)
NC_002695.1	alaW	3 244 939 – 3 245 014	3	75	75 (100.0)
	rrnG	3 446 266 – 3 451 276	322	5010	5010 (100.0)
	rrnD	4 158 428 – 4 163 775	341	5347	5347 (100.0)
	rrnC	4 735 252 – 4 740 264	321	5012	5012 (100.0)
	rrnA	4 831 654 – 4 836 758	328	5104	5104 (100.0)
	rrnB	4 975 927 – 4 980 939	322	5012	5012 (100.0)
	rrnE	5 016 953 – 5 021 965	318	5012	5012 (100.0)
	**total**		**2291**	**35 825**	**35 825 (100.0)**
					
*E. coli* str. K-12	rrnH	223 771 – 228 875	34	5104	5104 (100.0)
substr. W3110	aspV	236 931 – 237 007	0	76	0 (0.0)
accession:	alaW	2 523 602 – 2 523 677	0	75	0 (0.0)
NC_007779.1	rrnG	2 724 725 – 2 729 813	37	5088	5088 (100.0)
	rrfF	3 423 278 – 3 423 397	0	119	0 (0.0)
	rrnD	3 423 523 – 3 428 534	36	5011	5011 (100.0)
	rrnB	3 464 925 – 3 470 022	36	5097	5097 (100.0)
	rrnA	3 596 045 – 3 601 150	35	5105	5105 (100.0)
	rrnC	3 689 862 – 3 694 873	37	5011	5011 (100.0)
	rrnE	4 211 654 – 4 216 749	36	5095	5095 (100.0)
	**total**		**251**	**35 781**	**35 511 (99.2)**
					
*Phage Lambda*	Nim 290, 57, 60	41 053 – 42 370	**1179**	**1317**	**1317 (100.0)**
accession:					
NC_001416.1					

### Sensitivity and specificity of the method

There were 2522, 265 and 1232 total reads mapped on the reference genome of *E. coli* O157:H7, *E. coli* K12/W3110 and *Phage Lambda*, respectively. The majority of them (2291, 251 and 1179, respectively) were mapped on the intended enrichment targets. The sensitivity—the percentage of the target bases that are represented by one or more reads—and the specificity—the percentage of reads that map to the intended targets—of each individual experiment was 100.0% and 91.2% for *E. coli* O157:H7, 99.2% and 94.7% for *E. coli* K12/W3110 and 100.0% and 95.7% for *Phage Lambda*, respectively. The overall mean sensitivity and specificity of the method was estimated as 99.7% and 92.5%, respectively (Table 3).

### Performance of MinION sequencing runs

The mean and the maximum 2D read-length was 1769 bp and 5869 bp for *Phage Lambda* and 1908 bp and 15 645 bp for the *E. coli* experiments, respectively.

The mean percentage of identity of the 2D and the single-stranded (template) reads was estimated as 87.74% and 74.47% for *Phage Lambda*, 81.73% and 74.84% for *E. coli* O157:H7 and 82.44% and 74.85% for *E. coli* K12/W3110, respectively.

## DISCUSSION

The preparation of sequencing libraries enriched for specific target sequences for the MinION sequencing platform is challenging. In this study we describe for the first time a method that is capable of increasing the read coverage over the targeted sequences and their flanking regions, eliminating the majority of untargeted sequences. It is also designed to utilize platform-specific multiplexing primers (compatible with the PCR-adapters), which makes it applicable to multiple-target or multiple-sample sequencing studies.

The hands-on time of the described method (3–4 days) is significantly reduced in comparison with similar approaches (6–7 days) (10,11), through the parallelization of several steps, the reduction of hybridization periods and the use of commercially available reagents for the hybridization and the capture (see supplementary protocol outline). These, in combination with PCR-generated baits, provide a practical and affordable approach, in the same cost scale of the MinION device and its reagents.

The baits-probes that are commercially available can be used to enrich targets as large as complete exomes (>30 Mbp), while their size does not exceed 120 bp. In our study, the smallest average size of baits tested was 242 bp. These baits where more efficient on capturing the 5 Kbp fragments. At the same time, we show that the medium-sized—460 bp—PCR-generated baits were essential for the capture of longer DNA fragments, as the proportion of the 10 kbp fragments captured was 2-fold increased compared to those captured by smaller baits (Figure 2). Considering also the total yield of each enriched library, we used the medium-sized baits in our experiments. The generation of these baits via multiple PCR reactions is more affordable compared to the synthetic approach, but makes the method applicable to confined genomic regions. While the method is considered to be scalable, the laboratory workload would be increased significantly, due to the non-automated phase of baits generation. We successfully employed the method to enrich targets within the *Phage Lambda* genome but also within the 100-fold larger *E. coli* genome without any significant loss in performance. These data suggest that it can be easily adapted to other genomes of similar or larger size and complexity by the design of dedicated primers for the generation of the baits.

In order to take advantage of the extremely long DNA fragments that MinION sequencer is capable of analyzing, we optimized the shearing of the genomic DNA. We found that long—10 kbp—fragments were less likely to be captured, as the ratio of the targeted versus the untargeted copies of DNA was consistently lower, independently from the baits-length used. Reduced binding capacity may be due to steric hindrance developed around the streptavidin coated beads (12), a conclusion that is also supported by the relatively lower efficiency of the large-sized—∼900 bp—baits. This finding suggests that the usage of long baits and DNA fragments—greater than 5 kb—will need larger amounts of starting material, though the ratio of targeted to untargeted fragments is expected to be suboptimal.

The performance of the consumable MinION flowcells showed remarkable variance during the MinION Access Program (MAP) burn-in experiments, which was expected due to the evaluation phase of the platform and the ‘beta-testing’ of various chemistries and software packages that were gradually released from Oxford Nanopore Technologies. In our hands, the latest flowcells (chemistry R7.3) appeared to be more efficient and stable than the previous ones, although the flowcell that was used for the target enrichment experiment of Phage genome had only 272 (out of 512) nanopores active. This is also reflected in the final yield of the run, as we managed to take 11.5 Mbp as total output of the 48 h run. On the other hand, the flowcell used for *E. coli* run started up with 412 pores alive, resulting in a total output of 40.0 Mbp. The relatively low median read length of the 2D reads was expected as the shearing of the source DNA was on purpose aimed to relatively small sizes (5000 bp). The maximum 2D read length was comparable to current PacBio chemistry output (13).

The coverage of the targeted region of the *Phage Lambda* was significantly increased compared to the untargeted sequences. It must be noted that the overall yield and performance of the sequencing of the targeted regions might have been victimized due to the excess of the internal control in the final sequencing library and the resulting alteration of the availability of the nanopores. This effect was more intense in the enrichment of *Phage Lambda* rather than in the full-genome sequencing experiment, as the source DNA has undergone more processing steps than the internal control. The presence of reads corresponding to *Phage Lambda* control DNA was also intense in our *E. coli* experiments. Due to the existence of pro-phage elements all around the bacterial genome, these reads were affecting the coverage analysis and they were filtered out.

The yield of the barcoded reads mapping on rRNA operons of *E. coli* strain O157:H7 was higher compared to those mapping on operons of strain K12/W3110 (2511 versus 265 totally). Thus the sensitivity of the method was lower, failing to capture the smaller targets—off-operons tRNAs and solo 5S rRNA genes.

The enrichment performance was comparable with an other in-house method developed by Maricic *et al*. (10), which also uses PCR-generated baits, as we also managed to increase the read-coverage over the desired regions of the Lambda Phage and *E. coli* genomes. The main difference of our method is that we didn't use harsh conditions for the release of the captures from the baits. Instead, we amplified-released our captures in one step, using PCR and primers targeting dedicated, initially ligated, PCR-adapters. In the case of *E. coli*, these primers were hybrid, incorporating barcoding sequences and allowing for downstream pooling of the libraries and multiplexed analysis. This way, we can simultaneously release, amplify and add barcodes to the DNA fragments in a single PCR step, reducing the PCR bias introduced. This feature can be scaled up to 12 libraries, by the use of the compatible multiplexing primers, recently announced by Oxford Nanopore Technologies.

The estimated mean sensitivity of the method (99.7%) was comparable with the in-solution and on-array hybrid capture (>99.5% and 98.6%, respectively), while the estimated mean specificity was higher (92.5%) versus <80% and <70% for the same methods, as described in (6). This difference is probably due to the small-sized genomes tested in this study. It is important to state that these comparisons are only raw, given the scale difference in the DNA-fragments, the platforms’ outputs and the error rate, which affects the final mapping of the reads on the reference and is independent from the hybridization reaction itself.

Commercially available solutions for target enrichment (6,14) are not currently compatible with MinION sequencing libraries, as the chemistry of MinION sequencing-adapters is not compatible with these kits. Also, they have not been standardized for use with MinION's PCR-adapters and their biotin-baits (maximum length of 120 nt) have been optimized to capture shorter DNA fragments. Thus, although we would be able to use only the baits that are included in these kits, instead of producing them via PCR reactions, the capture of larger DNA fragments would be suboptimal, according to our findings (Figure 2). The baits could be also synthesized, which would be reasonable for smaller targets, up to 1–2 kb such as the one examined in *Phage Lambda* genome. For larger targets, the synthetic approach, although more convenient, would not be commercially affordable, compared to multiple PCR reactions.

Moreover, in our experiments we used the special Y-shaped PCR adapters and their compatible barcoding primers provided by Nanopore as they are natively recognized during the base calling and thus allow multiplexing of target enriched libraries. One alternative approach could use Transposase-5 (Tn-5) enzyme for simultaneous shearing and custom adaptor-tagging of the genomic DNA prior to hybrid capture, using the commercially available kits (15) but also in-house developed equivalent preps (16). The Tn-5 preparation kits are optimized for libraries with significantly smaller insert sizes (∼300–1100 bp) (17). A stoichiometric dilution of the enzyme in the reaction buffer, which is provided separately, could in theory deliver longer DNA fragments which would fall within the optimum sizes described in our study. Nextera's commercially available PCR handlers could then be used instead of MinION PCR adapters, but this would sacrifice the multiplexing capability of Nanopore's compatible primers. Using hybrid primers that would incorporate MinION multiplexing barcodes and at the same time be able to amplify the captured DNA fragments using Nextera's PCR handlers could provide an alternative solution. Another concern for this approach is the removal of the remaining Nextera sequences at the ends of the final reads which will either need to be identified before or manually trimmed after base calling. Extensive optimization experiments are needed to verify this conceptual method, especially with regard to the delivery of larger DNA fragments using Tn-5 preps.

Sequences flanking the target are commonly being captured during the enrichment process (18). Although these sequences are of small size, as the fragmentation of the source DNA and its size selection has always been based on the relatively small insert sizes and read lengths of the existing sequencing technologies, they have been successfully used for the identification of viral integration sites (11). During the preparation of MinION libraries the shearing of the source DNA is usually aimed at getting the longest possible fragment sizes. In this study we sheared the DNA at 5 Kbp, according to the finding that larger fragments are not captured efficiently, probably due to steric hindrance. The extended length of the captured DNA fragments resulted in more than 500 bp flanking the targeted area. The accurate remapping of partially off-target mapped reads to the same target-flanking regions during our BLAST analysis, further confirms that our method could be useful for structural studies—where the unknown neighboring sequences are of greater interest than the target itself—such as the identification of integration sites of viral elements. MinION long reads can also be informative for genome phasing studies assisting the scaffolding of genome regions that are difficult to be resolved, as recently reported (5,19). Thus, targeted sequencing of difficult regions could help in phasing of larger genomes.

## ACCESSION NUMBERS

High-throughput sequencing data have been deposited in the European Nucleotide Archive under the accession numbers ERR738435, ERR738436 and ERR855822 (Study accession PRJEB8285).

## Supplementary Material

SUPPLEMENTARY DATA
