# Supplementary material for: A novel method for the multiplexed target enrichment of MinION next generation sequencing libraries using PCR-generated baits
Source: Nucleic Acids Res. 2015 Aug 3;43(22):e152. doi: 10.1093/nar/gkv773 (PMC4678842; doi:10.1093/nar/gkv773)
Supplement: SUPPLEMENTARY DATA [file supp_43_22_e152__index.html]

A novel method for the multiplexed target enrichment of MinION next generation sequencing libraries using PCR-generated baits — A novel method for the multiplexed target enrichment of MinION next generation sequencing libraries using PCR-generated baits — SUPPLEMENTARY DATA 

# A novel method for the multiplexed target enrichment of MinION next generation sequencing libraries using PCR-generated baits

## SUPPLEMENTARY DATA

- SUPPLEMENTARY DATA
