# Supplementary material for: A novel method for the multiplexed target enrichment of MinION next generation sequencing libraries using PCR-generated baits
Source: Nucleic Acids Res. 2015 Aug 3;43(22):e152. doi: 10.1093/nar/gkv773 (PMC4678842; doi:10.1093/nar/gkv773)
Supplement: SUPPLEMENTARY DATA [file supp_gkv773_nar-00170-met-k-2015-File010.pdf]

Supplementary Material

Supplementary Figure 1

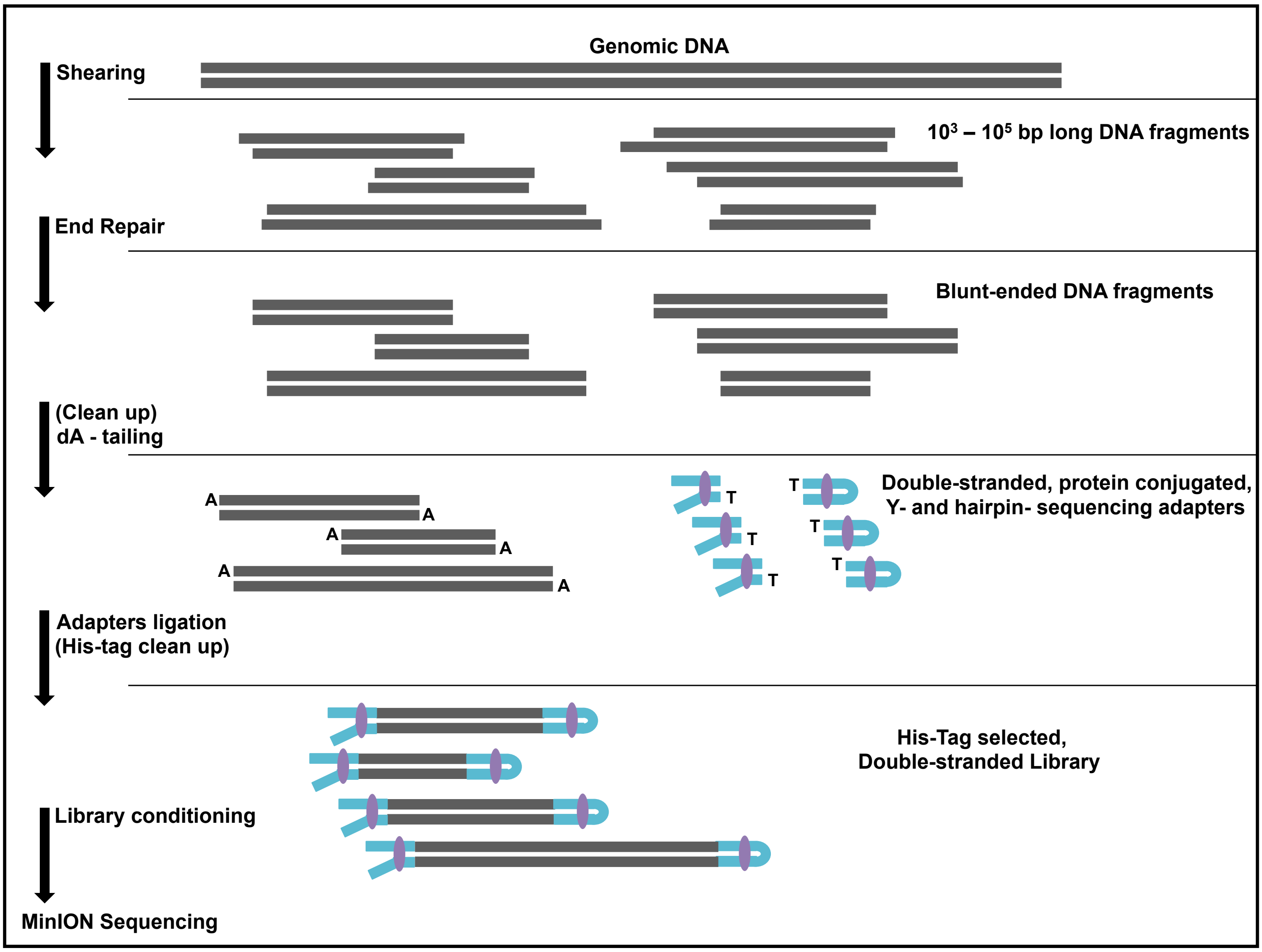

MinION's standard protocol for genomic DNA library preparation. The procedure shares many common steps (DNA-shearing, end-repair, dA-tailing etc) with other well-known platforms. Crucially, the length of the DNA fragments is extremely long (10^3 – 10^5 bp) compared to other platforms. The sequencing adapters (in green) are specially shaped to form hairpin- and Y- shaped double stranded molecules. Platform-specific proteins (Motor enzyme, HP motor, Tether - in purple) are conjugated on these adapters allowing control of the speed during the translocation of the strands through the nanopores and promoting saturation of the fragments on the sensor chip. A final His-tag purification step enriches the library for double stranded molecules, as they are selected based on the existence of the hairpin adaptor. The "pre-sequencing mix" has to be conditioned (mixed with the "fuel mix" and the "EP-buffer") directly before each loading on MinION's flowcell.

Supplementary Figure 2

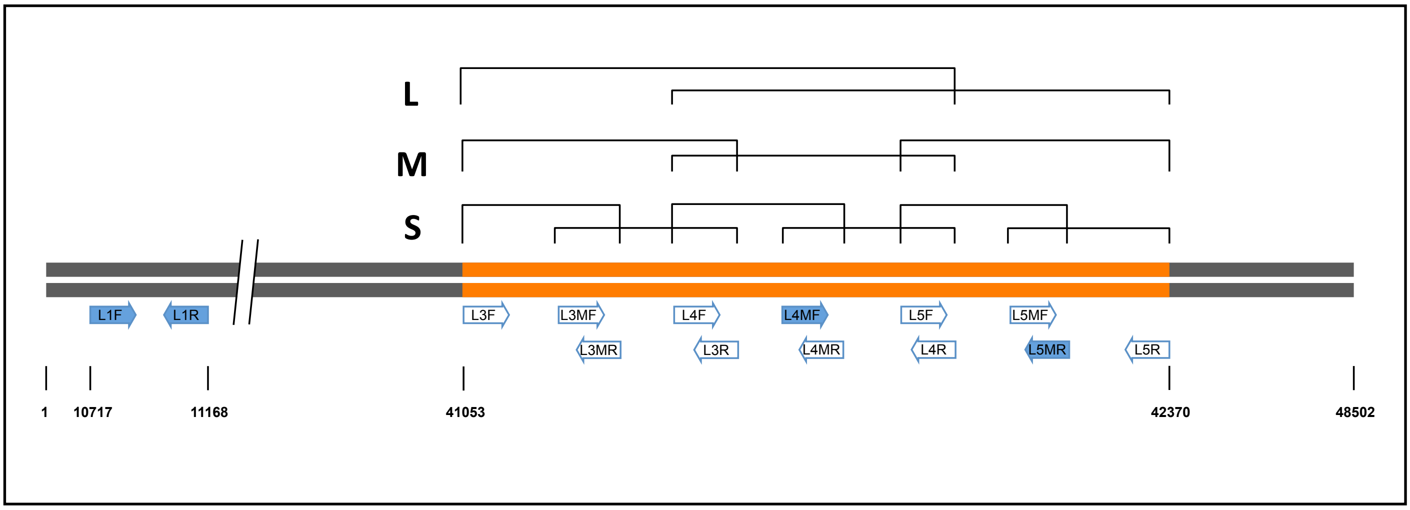

Schematic representation of the baits used for the optimization of the method. The combination of 6 forward and 6 reverse primers (outlined in blue) resulted in 2 large (L), 3 medium (M) and 6 short (S) PCR-generated baits. These 3 pools were targeting the same genomic region of phage lambda genome (orange bars). Solid blue arrows indicate the primer sets used for the assessment of the enrichment procedure. The PCR product of primers L1F and L1R corresponds to an untargeted region of the genome, which was used as a control.

Supplementary Figure 3

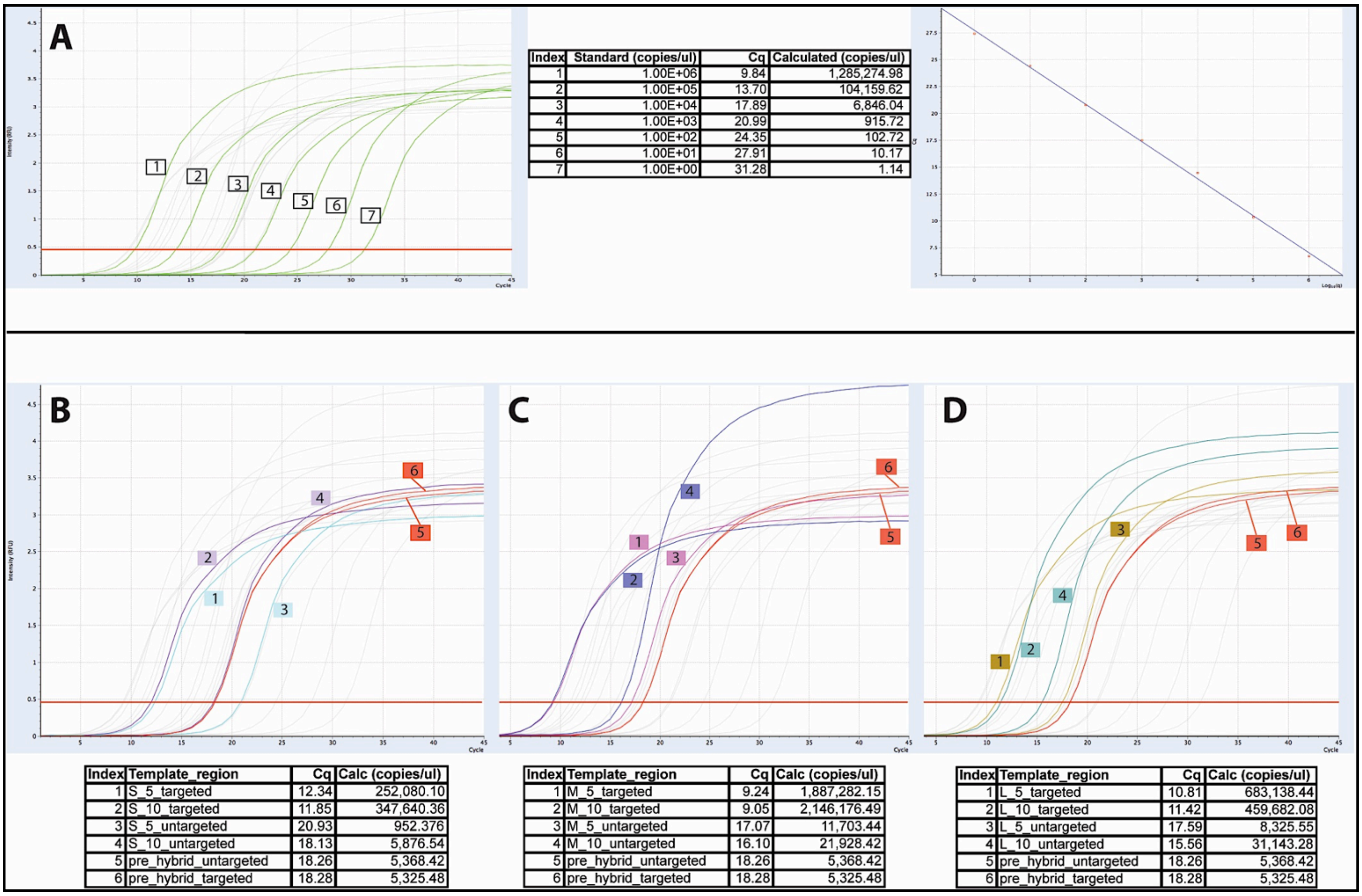

Index	Standard (copies/ul)	Cq	Calculated (copies/ul)
1	1.00E+06	9.84	1,285,274.98
2	1.00E+05	13.70	104,159.62
3	1.00E+04	17.89	6,846.04
4	1.00E+03	20.99	915.72
5	1.00E+02	24.35	102.72
6	1.00E+01	27.91	10.17
7	1.00E+00	31.28	1.14

Index	Template_region	Cq	Calc (copies/ul)
1	S_5_targeted	12.34	252,080.10
2	S_10_targeted	11.85	347,640.36
3	S_5_untargeted	20.93	952.376
4	S_10_untargeted	18.13	5,876.54
5	pre_hybrid_untargeted	18.26	5,368.42
6	pre_hybrid_targeted	18.28	5,325.48

Index	Template_region	Cq	Calc (copies/ul)
1	M_5_targeted	9.24	1,887,282.15
2	M_10_targeted	9.05	2,146,176.49
3	M_5_untargeted	17.07	11,703.44
4	M_10_untargeted	16.10	21,928.42
5	pre_hybrid_untargeted	18.26	5,368.42
6	pre_hybrid_targeted	18.28	5,325.48

Index	Template_region	Cq	Calc (copies/ul)
1	L_5_targeted	10.81	683,138.44
2	L_10_targeted	11.42	459,682.08
3	L_5_untargeted	17.59	8,325.55
4	L_10_untargeted	15.56	31,143.28
5	pre_hybrid_untargeted	18.26	5,368.42
6	pre_hybrid_targeted	18.28	5,325.48

Quantitative assessment of the enrichment process. Two Phage-Lambda regions, one targeted and one untargeted were amplified using as template genomic-DNA (pre-hybridization control) and 6 enriched libraries. (A) Screenshots of qPCR standard curve data. Enrichment using: (B) Baits of mean length 242bp (short-“S”), (C) baits of mean length 460bp (medium-“M”), (D) baits of mean length 884bp (long-“L”), in combination with 5Kbp (“5”) or 10Kbp (“10”) genomic DNA shearing size, all compared to the pre-hybridization genomic DNA amplifications (red curves). Threshold value: 0.47 (horizontal red lines).

Supplementary Figure 4

IGV screenshot showing reads mapping on the Phage Lambda target. Randomly selected reads (in red, blue, brown, purple, green) that span the target coordinates have been analysed with BLAST and the respective alignments are presented in the following figures (4A-4E, read names encoded by colour). Alignments corresponding to target and to flanking sequences are in light yellow and light blue, respectively.

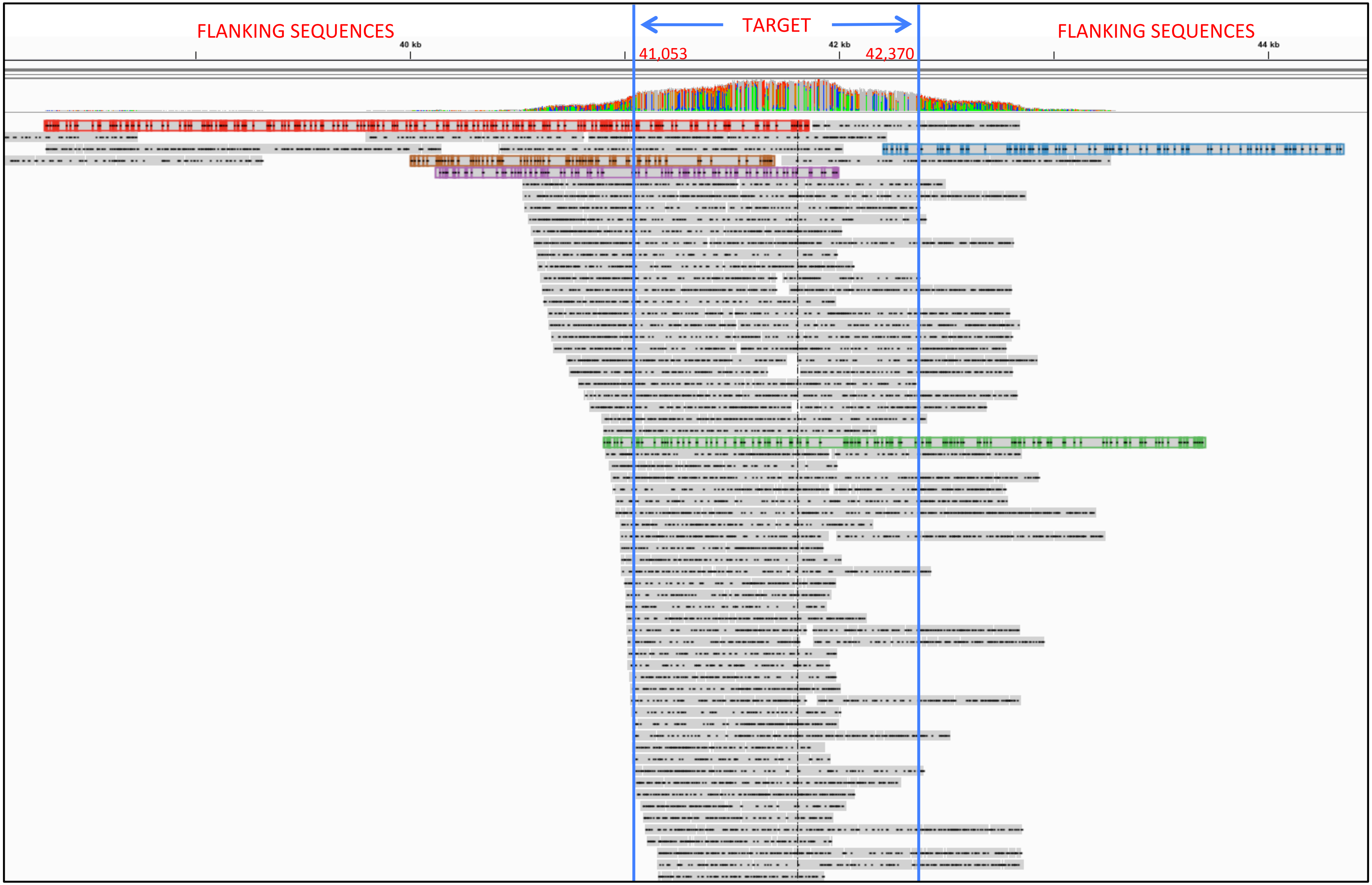

4A. Read_name_channel_73_read_49

Score	Expect	Identities	Gaps	Strand
4044 bits(4484)	0.0	3123/3541(88%)	247/3541(6%)	Plus/Plus

```
Query  62     TTCGTGCAAAACAACGCAACGAGGCTCTACGAATCCAG-GTGCGTTGCTTAACAAA-TCGCAATGCTTGGAACCAAAGAACACAGCG-AAGCTGTGGGCGTTGATAAGTCGCAGATCAGCAGGTGGAAGAGGGACTGGATTCCAAAGTTC  208
Sbjct  38364  ..........CA.......................G..A.................A................TG.-...G......G..............................................................  38512

Query  209    TCAATGCTGC-TGCTGTTCTTGAAATGGGGGTCGTT----ACGACACGGCCTCGATTGGCGCGACAAGTTGCTGCGATTCTCACCAAT-AAAAACGCCCGGCGGCCCAACCG-GCGTTCTGAACCAAATCCAGATGGAGTTCT-CGATCA  350
Sbjct  38513  ..........T.............TG..........GACG.......T.-......................................A...............--......A..........-...................GA.G...  38658

Query  351    TTACTGGATCTATCAACAGGATGGCTTTA-AACAAATACAGC--AAATACTCAA-TTCGGCAGAGGTAATTTATCAGGGACACAGGGAGCGTAATGTGGCAGATCTCGATGATGGTTA-ATCAGACTATCAAATATGCTGC-TGAGGCTG  494
Sbjct  38659  ......................-.T.A...TG..........AA..........C..............C..-.GCC.....---................................CGC.....................T.......T  38803

Query  495    TCACGGGCGCAGATCTGACCAAGGGACAGTTCAAAGTGCTGCTTGCTCATTCTGCGTAAAACCTATGGGTGTACGAATAAACCAATGGACAGAATCACCGATTCTCGACTCAGCGAGATTACAAAGTTGGTCTGTCAAACGGTGCAATGA  644
Sbjct  38804  ATT....................C.......T..............-........................---................................A...T................-AC....................  38948

Query  645    AGCCACAGTCGTAGAACTCGTCAGAAGATGAATATTATGACAAGCAGCAAGCGGGCATGTTGGTAACCAACATAAACATCTCAGAATGGTGCCAATCCCTC-AAACGCTAGAAA--TCCCTAAAACTCGAGGGAT-AAACATCCCTCAAA  790
Sbjct  38949  .....-..--...............--...........--...........GC........T.G.C.A..T.A...................--.......A.....AGG....ATC........--........A..............  39087

Query  791    TTGCGGGA-TGCTATCCCTCAAAACA-GGGGAC---AAAGAC---ATTAC-----AAAGAAGAAAAGATTATTCGTCAGGGAATTCTGGCGAATC--CTGACCAGCCAG--AACGA-CTTTCTGTGGTG-AATGAGATGCTGGCACTAAT  921
Sbjct  39088  ...G....T.................G......ACA......ACT.....AAAAG...A.....................A..............CT............AA.....C............A..CCG.......----C...  39233

Query  922    TC--AGCGGCAGCAAGTGGGGGACAGCAGAAGACCTTA-CGCCGCAGTGAGGATGTTTGGCATGGCGAAGACTATCGCACCATCAGCCAGAAAACCGAA-TTTGCT-GGTGGGGTAGACCGATATCCGCCTGAT-CGTGAACGTGACTAA  1065
Sbjct  39234  ..AG................................G.C........A.T.........A....T..................................T......G......C.--.A...............G............GG.  39381

Query  1066   CGTAACCACCGCGACA--TGTGTGCTGTTCCGCT-GGCGTGCCAGGACAATTTC-GGTCCGGTAACGTGCTGAGCCC-----AACCCTGGCCCAATAGTACTCCA-TCGCAATCAACCGTAACAAGCAAAGCGGCAGGCGTGACAGCCAG  1205
Sbjct  39382  ................TG................G...A...........C...T......................GGCCA...T.C.CGAT..GT.G..C.A.C...A.................-..CA..................  39530

Query  1206   CAAACC--GACTCGACCTGACAAACACAGAC-GGATTTACGGGGTGGATCTATGAAGACACTCATCG-CGCACAGATGGTTAACTTTGACCGTGAGCAGATGCGTCGGATCGCCAACACAACATGCCGGAACAGTACGACGAACAAGCCG  1351
Sbjct  39531  ......AAA......................T........................---.AA.....C...............................................--...........................-.....  39674

Query  1352   CAAGTACAGCAGGTA--GCAGATCATCAACGG-GTGTTCAGCCAGTTAATGGCAACTTTCCCGGCGAGCCTGGCTAACCGTGACCCAAGAACGAAGTGAACACGAAATCTTTCGCCAGTAGGGTTCTGGCTTTTCGGGGAAACGGGAAT  1498
Sbjct  39675  ..G............GC...............T...............C..........................................--.........--.......CG.......-...............-...A.......TC  39818

Query  1499   A-CACGTATGAACACCATTAACGCAGGAATGCG---GGCCCGTTGGCAGGATCGACCATTTTCTGCCATCACCCGGGCAGTTTGTTGCATGGTGCCGGGAAGAAGGAGCATCCGTTACCCGCCGGACTGCGCTCAAACGTCAGCGAGCTG  1644
Sbjct  39819  .C....ATG.....-GG................CGTA......C.....A.........-...........................................---...........-............---.................  39959

Query  1645   GTTGATATGGTTTACGAGTATCACTTAGAAGCGAGGCCTGTATCGGCCACTGCGGAGTCTTATCCGTGTGAAATC-AACGCGCACTACTGGCTGGTTACCAACCTGTATCAGAAC-TGCGGGCCAATGCGCTTACTGATGCGGAATTACG  1792
Sbjct  39960  .....................TG.-CG.................--.GGA..................-......A.......................................A..................................  40105

Query  1793   CCGTAAGGCCGCAGATGAGCAAGGTCCCCATATGACTGCGAGAATTAACCGTGGTGAGTAGGCGATCCCTGAA-CAGT-AAACAACTTCCTGTCATGGGCGGTAGACCTCTAAATCGTGCACAGGCCTCTGGCGAAGACTGC--AAATCA  1938
Sbjct  40106  ....................---T.GT.............................---..............C....A............................................-..............TC..AG......  40248

Query  1939   AAGCTAAGTTCGGTACTG-AAGGAGCAAGTGTATGACGGGCAAAGAGGCAATGTCATTCATTACCTGGGGACGGCATAATAGCTTCTGTGCGCCGGACGTTG-CGCGCTAACAGGCGCAAACAGTAACCGGCATAATAAATCAGGCGCGT  2086
Sbjct  40249  .............-....A...............................--.T..................-.........................C.................-..........A..---.............----  40386

Query  2087   ACTGGGCTAAAATGGCACGTGGCAGGTCTTCTGGTTATCGAAGGTAAGGTC-GGCGAACGGTGTATTACATCATTTGCTACCAGGGAAGAACGGGAAGG-AAGATGAGCACGAGCAACCTGG-TTTTAAGGAGTGTCGCCAGAGTAGTGC  2233
Sbjct  40387  -.GC..............-................................T.................-CGG..........................A...........---........T...................---.....  40527

Query  2234   CG-GCTGAAACGGGTATTGGCGGTATAT-GAGTT-AAAGATGACCATCTAAGCTTACTGAGCTAATAACAGGCCTGCTGGTACGGGCAGGCC-TTTGATTT-GGGGAGAGGGAAGAGTCATG--AAAACTAACCTTTGAAATTCGATCTC  2376
Sbjct  40528  ..C.A.......................G.....A..................-.CA.........................ATC.......T...T...G...........--.......AA...........................  40674

Query  2377   CAGCACATCAGCAAAAGGCCTATTCACGCAGTACAGCAAATCCTTCCAGACCTACAACCAAACCAATCGCAGTAACCATTCAGGAACGCAACCGCAGCTTAGACCAAAACAGGAAGCCTATGGGCCTGCT--GCTTATGTCTC-CTTC-G  2522
Sbjct  40675  ................C.-.................................--...............T..............................................-.............TA.G.G.C.....T.G..A.  40820

Query  2523   ATTGAGTGAAATGGTCGCTGGCTGGATGCAGAGAAAGCTGGAA--GTGTGTTTACCGCAGCATTAAAGCAGCAGGCCATGTTATTTCCTAACCCTTGCCGGGGAAACCCGCGCTTTGT-----ACCTCCAG---ACCAGCAGGATGCGTG  2662
Sbjct  40821  G....A..GC....................--...........GT...............................--.....-G......-..........A.-----TG.......GGTAATAGG....TCA................  40959

Query  2663   TAGGCGTTAAATTTGCGACTATATTAGAGCTTATACAAGGCATTCGGTACAGCGAGACGCGTGGCGTTAA-TGGTCAGACGAAGC--GACTGGCTCTGGAGTGGAA---GAGAT-GGAAGACAGGGCTGCATGATAAATGTCGTTAGTTT  2805
Sbjct  40960  .....---.........GAGC..............-..................-----A...........G.............GA...................AGC.....G..G................................  41100
```

<FLANKING 41053 TARGET>

```
Query  2806   CTCCGGTGGCAGGACGTCAGCATA-TTGCTCTGGCTAATCGAGCAAAGCGCGACGGGGCAGGTAACACTTTGCATTACG-TTTCATGGATACAGGTTGTGAACATCCAATGACATATCGGTTTAT--TGGAAGTTGTGAAGTTCTGGGGA  2951
Sbjct  41101  ........................T..............G.......-A.....-.........AGACG..........T...........................................G.CAG.................-....  41247

Query  2952   TATACCGCTCACCGTATTGCAGGTTGATATCAACCCGGAGCTTGGACAGCCAAATGGTTATAC-GTAT-GAAACCAAAGGATATTC-GACGGGAACTGCCTGTTCTGAAGCCATTTATCGATATGGTAAAGAAATATGGCATTC--TAGA  3096
Sbjct  41248  ...............................................................G....G.G...............A....C..-..............................................C..CA..CG  41396

Query  3097   TTACTAGCGCGTTCTGCAATGACAGATT-AGACTCGTTCCCTTCACCAAATACTGTGATGACCATTTCGGGC--GGGAA-TGCCCTACGT-GATT-GCATCAGAGCTGATGAACCGAAGCGGCTAAAGCCAAAGCCTGGAATCAGATATC  3240
Sbjct  41397  .CGGCG...........C..........A.A.........................................GA.....T.A.A.C....G....G......................................................  41546

Query  3241   TTGCTGAACCTGTCAGACTTTGAGAAGGAGGATATCCTCACATGGTGGAAGCAACAACCATTCGATTTGCAAAATACCGAACACATCTCGGTAACTGCATATTCTGCATT-AAATATCAACGC--AAAATCGAAGACTTGCCTGCAAAGA  3387
Sbjct  41547  .......-.....................A........G...............................-........G.-............................A...A........AA......--.................  41691

Query  3388   TGAGGAGGGATTGCAGCGTG-TTTTAATGAGGTCATCACGGGATCCCATGTGCGTGACGGACATCGGGAAACGCCGAAGGAGATTATGTAC  3477
Sbjct  41692  ....................T......................................................A...............  41782
```

4B. Read_name_channel_450_read_17

Score	Expect	Identities	Gaps	Strand
2424 bits(3260)	0.0	1918/2098(91%)	143/2098(6%)	Plus/Plus

```
Query  69     TTGACCAAATTGGCACGAT--GAACAAGAAAGCGTCGAGCG--CTGTTAGACGTGCGCTAACTGCGGTCAGAAGCTGCATGTGCTGGAAGTTCACGTGTG--AGCAATGCTGCGCA--ACTGATGAGCGATCCGAATAGCTCGATGCACG  210
Sbjct  42273  .........A.C.A.-.T.AC....................AG..-...-..................................................TG....C.........GA................................  42419
```

<TARGET 42370 FLANKING>

```
Query  211    AGGAAGAAGATGATGGCTAAACCAGCGCGAAGACGATGTAAAA-CGATGAATGCCGCGGGAATGGTTTCATGCCCCTGCATTCGCTAATCATCCCTG-TGGTGCTTCTCCAGAATGTGGAACCAAGATAGCACTCGAACGACGAAGTAAA  358
Sbjct  42420  ...........................................A............--............---..................G---..G.......-.......G....................................  42560

Query  359    GAACGCGAAAA-GCGGAAAA-GCAGCAGAGAAGAGA--ACGACGAG-GGAGCAGAAACAGAAAGATAAACTTAAGATTCGAAAACTCGCCTTAAAGCCCCGCAGTTACTGGATTAAACAAGCC-AACAAGCCGTAAACGCCTTCATCAGC  502
Sbjct  42561  ...........A........A.............A.CG........A............................................................................C.........................A  42710

Query  503    GAAAGAGACCGCGACTTAAGTTGTATCTCGTGCGGAACGCTCACGTCTGCTCAGTGGGATGCCGGACATTACCG---AACTGCTGCCGGCACCTAACTCAACTCCGATTTAATGAACGGGAATATTCA--AGCAATGCGTGGTG--CA-C  644
Sbjct  42711  ..................CCA.....................................................GAC.........-.......----....................C-........CA..............TG..A.  42854

Query  645    CAGCACACAC-CGGAAATCTCGTTCCGTATCGC-TCGAACCGCACTAGCCGCATGCCGGGCAGGAAGCAGTAGACGAAATCGAATCCGTCAAACCATAACCGGG-TCCC-GGACCTATCGACAGAGTGATGCAAGGCGATCAAGGCAGAG  790
Sbjct  42855  .......A.AG......................G......T.-.T.........--..............................----............CCA..G.T..-........-.....---....................  42992

Query  791    TACCAACAGAAACTCAAAGAAGCTGCGAAATAGCAGAA-TGAGGCCGCATGACGT-CTCAGTAAAA-CCATTCGAGACATGCTCGTTGAAACATACGGAAATCAGACAGAAGAACAAATAGCACGCAGACTGAAATCTAGTCGCGGTACG  937
Sbjct  42993  ....................C-................G................T..........A......C................G.....................------..................G.............  43135

Query  938    GTCAGAAAATACGTTGATGATAAAGACGGGAAA-TGCACGCCATCGTCAACGACGTTCTCGATGGTTCATCGCGGATGGAGTGAAAGAGATGCGCTATTACGAAAA--TTGATGGCATAAGCAAATACCGAAATATCATTTGGG--GCTT  1082
Sbjct  43136  .................................A..........................-.............................................AA.........---...............---......TA.-..  43277

Query  1083   G---ATTTGCACGGATGCTACACGAACCTGAGATGAACAA-C-GGATACGATTGGATTCGACAACAAAA--GACCTGCTTATCTCGGTGGGCGATTTGGTTGATCGTGGTGCAGAGAACGTTGAATGCCTGGGAATTAATCACATTCCCC  1225
Sbjct  43278  .GCG..C........................--.......A.T..........................AA.............................................................-.................  43424

Query  1226   TGGTTCAGAGCTGGTACGTGGAAACCATGAGCAAATGATGATTGATGGCTTATCAGAGCGTGGAA-CGTTAATGTACTGGCTGCTTAATGGCGGTG-CTGGTTCTTTAATCTGGGCGATTACGACAAAGAAATTCTGGCTAAAGCTCT-G  1372
Sbjct  43425  .............-...................................................A.......C-.....................G...............---.................................T.  43569

Query  1373   CCCATAAAGCAGATGAACTTCCGTTAATCATGGCAAC-GGTGAGCAAAGAT--AAAATATGTTATCTCTGCCACGCCGATTATCCCTTTGACGAATACGAGTTTGGAAAGCCGGTTGATCATCAGCAGGTAATCTGGAACCGCGAACGAA  1519
Sbjct  43570  ...............................C.-...T.............AA...........--..............................................A.....................................  43716

Query  1520   TCAGC-ACTCACAAAACGGGATCCTTGAAAGAAATCAAAGAAGCGGACACGTTCATCTTTGGTCATACG-GAGCAGTGAAACCACTCAAGTTTGCCAACCAA----ATATCGATACCGGCGCATTGTTCTGCGGAAACCTAACATTGATT  1663
Sbjct  43717  .....A..................-.G..............GC.........................CC................................ATGT.................G..........................  43865

Query  1664   CAGGTACA-GGAGAAGGCGCATGAGACTCGAAAGCGTAGC---ACTTCATTCGCC--AAAGCCCGATGATGAGCGACTCACCAC-GGCCACGGCTTCTGACTC-CTTTCCGGTACTGATGTGATGGCTGC--TGGGGATGGCGCAATCAC  1803
Sbjct  43866  ........G...............................TAA.T..........AA...........................G..................T..........................TA..................  44015

Query  1804   AAGCCGGATTTCGGT-TGGCTGCATTCTGCGGTAAGCACGAACTCAGCCAGAACGACAAACAAAAGGCTAGTAAACGTA--TGATGCAATTTGCACACACAAGGTATCGGGG-AATACCGTGGGTGTGGGAAAGCTTGAAGGAAGTACTA  1949
Sbjct  44016  ........-......A.......................................................-.C..-..TC...........--..................A........-.......C..............A.....  44159

Query  1950   AGGCGGCAAA-GTACTGCAAGTGCTCGCAACATTCGCTTATGC-GATTATTGCCGTAGTGCCGCGACGCC-GGGGCAAGATGCAGAGATTGCCATGGTACAGGCCGTGCGGTT--TACTTGCC-AAACAGAGCTGT-GGGGAGAGTTG  2090
Sbjct  44160  .---......G................................G..........................G..........................................GA..-.....A............G...........  44303
```

4C. Read_name_channel_144_read_4

Score	Expect	Identities	Gaps	Strand
1975 bits(2190)	0.0	1465/1642(89%)	101/1642(6%)	Plus/Plus

```
Query  42     TGGCTGGTTACCAACCTGTATGTCAGAACATGCGGGCCAATCGAGCTTACTGATGCGGAATTACGCTGTAAGGCCGCAGATGAGCTGTTGTCCAT--GACTGCGA--ATTAAGACCGTGGTGAGGCGAATCCCTGAACCAGTAAA-CAAC  186
Sbjct  40044  .....................--..................-.C......................C...................--.......AT........GA....--...............-................A....  40185

Query  187    TTCGTGTCATCTGGCGGTA--CCCCCAAATCGTGCACAGGCT--GGTGAAGATCGCAGAAATCAAAGCTAAGTTCGGACTGAAAGGAGCAAGTGTATGACGG-CAAAGAGGCGCAATTCCATATTCATTAC-TGGGGACGCATA----CT  326
Sbjct  40186  ...C......G-.......GA..T.T................CT..C.......................................................G........--.....----.........C............ATAG..  40328

Query  327    CCTGCGCGCCCTAACGTTGCCACCCTAACAGGCGCGAAACAGTAACCAGCATAAATCACCCACGCGGCTAAATGGGGACGGGCAAGT--TCTGGTG--CGA--GTAAG-TCTTGAGAACGGTGTATTACCGGTTTGCTACCAGGGAAGAA  469
Sbjct  40329  T...T.....GG.-.......G.G...........--.....................GG.-..........AT..C.......G..CT......TAT...AG.....G...G.C...................................  40474

Query  470    CGGGAAGGAAAGATGAGCACGAACCTGGTTTTTAAGGAGTGTT--CAGAGTGCCGCGATGAA-CGGGTATTGG-GGTATATGGAGTTAAA-GATGACCATCTACATTACTG-GCTAATAACAGCCTGGCTGCTGGTATCCCAAGGC--TT  611
Sbjct  40475  ..........................................CGC.................A..........C................A....................A...........G.---.........AT.GC....CT..  40621

Query  612    TGATTTGGGGGAGAGGGAAGTCATGAGACAAACTAACCTTTGAAATTCGATCTCCAGCACATCATAGCAACGCTATTCACGCAGTACAGCAAATCCTTCCAGACCCAACAATA---ATC----TATCCATTCAGGAACGCAAACCGCAGC  754
Sbjct  40622  .T........................A.-...................................GCAA.........................................C.A.CCA...GTAG..A................-.......  40769

Query  755    TTAGACAAACA-AGGAAGCTATG---CTGCTTAGC-GACCT-TCTCGTCAG-TTGAATGGCATGGT-GCTGGCTGGCTCATGCAGAA-GCTGGAAGTGTGT--TTACCGCAGCAGATTAAAGCAGCAGGATGTTGTTCCTAACCTTGCCG  893
Sbjct  40770  ......C..A.C...........GGC........GT...G.C.........G..............C.........---........A.............GT...........--..................................  40914

Query  894    GGAATGGCTTTGTGGTAATAG--CGGTCAACCAGC-GGA-GCGTGG-GGCGAATTTGTCCAGCTATTAGAGCTTATACAGGCATTCGGTACAGAGCGGTGGCGTTAA-TCGTCAGACGAAGCGAGACTGGCTCTGGAGTGGAAGGC--GA  1035
Sbjct  40915  .....................GC.A..........A...T.....TA..........CGG....................................-..........G.G.................................A..GA..  41063
```

<FLANKING 41053 TARGET>

```
Query  1036   TGGGGA-AAGCGGCTGCATGATAAATGTCGTTAGTTTGACGCTGTGGCAGGACTTTACTACCATATTTGCTCTGGCTAATGGAGCAAAGCGAGACGGGCAGGTAACACGGTGCATTACGTTTTCATGGATACAGGT--TGGACATCCAAT  1182
Sbjct  41064  ......G.CAG..........................CT.-.G..........G.---C.G............................-A.C............AGAC...........................TG..A.........  41208

Query  1183   GACATATCAGTTTGTCAGGGAAGTTGTACAGTTCTGGGATATACCGCTCACCGTATTGCAGGTTGATATCAACCCGGAGCTTGGACAGCCAAATGGTTATACGGTATGGGAACCAAAGGATATTCAGACGCGAATG-CTGTTCTGGTGAC  1331
Sbjct  41209  ........G..................GA...........................................................................................................C........--A.G  41356

Query  1332   CCATTTATCGATATGGTAAAGAAATATGGCACTCCATACGTCGGCGGCGC-TTCTGCACTGACAGATTAAAACTCGTTCCCTTCACCAAATACTGTGATGACCATTTCGGGCGAGGGAATTACACCACGTGGATTGGCATCAGAGCTGAT  1480
Sbjct  41357  ..................................................G...................................................................................................  41506

Query  1481   GAACCGAAGCGGCTAAAGCCAAAGCCTGG-ATCAGATATCTTGCTGAACTGTCAGACTTTGAGAAGGAAAGGTAACACCGCATGGTGGAAGCAACAACCATTCGATTTGCAAATACTAGGACATCTCGGTAAACTGCATATT  1621
Sbjct  41507  .............................A.....................................-...A..T.CT......................................CG.A..........-...........  41646
```

4D. Read_name_channel_95_read_12

```
Score               Expect   Identities         Gaps             Strand
2221 bits(2462)     0.0      1663/1869(89%)     120/1869(6%)     Plus/Plus

Query  53     AAACAACTTCCTGTCATGGGCGGGT-ACCTCTACGGTAAATTCTAGCACAGGCT--GGCGAAGATCGCAGAAATCAAAGCTAAGTTCG-ACTGAA-GGAGCAAGTGTATGACGGT-AAAGAGGCAATTATTCATTACCTGGCTACGCATA  196
Sbjct  40179  .......................TAG.......------.A..GT.........CT................................G......A..................GC.........................GG.......  40322

Query  197    ATAGCTTCTGTGCGCCGGGACGTTGCCGCGCTAACAGGCGCAACAGTAACCAGCATAAATCAGGCCGCGGCTAAAATGGCCCGGCGGGTAGGTCTTCTGGTTATCGAAAGTAAT---TGGCGACTTAGCTTATTACCGGTTTGCTACCAG  343
Sbjct  40323  ..................-.............................................................A..---.C....................G....GGTC......ACG-.TG....................  40467

Query  344    GGAAGAACGGAGGGAAGGAAAGATGCT-ACGAACCTGGTTTT-AAGGAGTGTCTC--GAGTGCCGCGATGAAACGGGT-TTGGCGGTATATGGAGTTAAA-GATGACCAACTACATTACT---CTATAGATAACAGGCCTGCTGGTAATC  484
Sbjct  40468  ..........---............AGC..............T..........G.CA.....................A.....................A.........T..........GAG...---....................  40611

Query  485    GCATAGGCCTTTT-ATTTACCAG-GAAGGAAGTCAGTAGCAAAAACTAACCTTTGAAATTCGATCTCCAGCACATCAGCAAAACGCTATTCACGCAGTACTAAGCAAATCCTTCCAGACTCCAACCAAACCAATCGTAGTAACCATTCAG  632
Sbjct  40612  ...--........T....GGGG.A..G.........TG.--............................................................--.................-.............................  40754

Query  633    GAAACGCAACCGCAGCTTAGACCACAAACAGGAAGCCTATGGGCCTGCTTAGACCTTCTCCCACGTCGGGTTGAATGGGGAGCGCATGGTCGCTTACGATGGGACTGCAGAAAGCTGGAAGTGTGTGTTTACCGCAGCAGATTAAAGCAG  782
Sbjct  40755  ...-....................-...........-...............GTGACG..T.T....A..........------..........GG.T---...-..................................--.........  40889

Query  783    CAGGAGATGTTGTTTCCTAACCTTGCCGGGAATGGCTTTGTGGTAATAGGCCAGTCAACCAGCAGGATGCGTGTAGGCGAATTTGCGGAGCTATTAGAGCTTATACAGGCATTCGGTACAGAGCGTGGCGTTAAGTGGTCAGACGAAGCG  932
Sbjct  40890  ....--........-.......................................................................................................................................  41036

Query  933    AGACCTGGCTCTGGAGAGTGGAAGCGAGATGGGGA-ACAGGGCTGCATGATAAATGTCGTTAGTTTCTCCGGGCGGCAGGACGTCAGCATATTTGCTCTGGCTAATGGAGCAAAAGCGACGGGCAGGTAAAGACGTGCATTACG--TTCA  1079
Sbjct  41037  ...-............T.-.A..............G..................................-..T......................................................................TT....  41183
                              <FLANKING 41053 TARGET>

Query  1080   TGGATACAGGT--TGAACATCCAAGGTGACATATCGGTTTGTTACACGGAAGTTGTGAAGTTCTGGGATATAAGCCTCACCCGTATTGCATTATGATATC-ACCCGTATAGC-TGGACAGCCAAAATGGTTAACCGGTATGGGAATCAAA  1225
Sbjct  41184  ...........TG...........--................--..G.........................CCG...-...........GGT........A....--G...T.......-...........TA...........C....  41325

Query  1226   GGATATATTCAGACGCGATTACGCTGTTCAACGAGCCATTTATCGATATGGTGACAGAAATATGGCATTCCATACGTCGGCGGCGCGTTCTTAAGGTGACAGATGCTAAAACTCGTTCCCTTCACCAAAATACTGTGATGACCATTTCGG  1375
Sbjct  41326  ..--..............A.G.-.......-TGA...................-.A...........C.......................GC.-C.........--...................-.......................  41466

Query  1376   GCGAGGGAATTACCACCACGTGGATTGGCATCACCAGCTGATGAACCCGAGCGGCCTCAACAAGCCAAATGCCTGGAATCACATATCTTGCTGACCTGTCAGACTTTGAGAAGGAAAGATATGCGCCCGGGACTTGGAAGCAACAACCAT  1525
Sbjct  41467  ............-....................-G............GA.....----TA.........-...........G.............A..................-.......C.T.G.AT.--G................  41606

Query  1526   TCGATTTGCAAATATACCGAACATCTCGGTAACTGCATATTCTGCATT---AAATCAACGC---AAATCGGACTTGCCTGCCAAAGATGAGGGAGGATTGCAGCGTG--TTTAATGAGGTCATCACGGGATCCCATGTGCGT-AGGGACA  1666
Sbjct  41607  ..............-C.G..............................AAA..........AAA................-...........AG.............TT.................................G.C.....  41754

Query  1667   TCGGGAA--GCCACGGGAGATTATGTACCGAGG-AGAATGTCGCTGGACATGTATCTCGCGAAAATGTATTCAGAAAATGATTATCAAGCCCCGTATCAGGACATGGTACGAGCT-AAAGA-TCGATACCGGCTCTTGTTCTGAGTCATG  1811
Sbjct  41755  .......AC....AA..................A...............-G..--.....................................T......................A.....T............................  41901

Query  1812   CGAAATATTTGGAGGGCAGCTGTGATTTCGACTTCGGGGAGGGAAGCTGCATGATGCGATGTTATCGGT  1880
Sbjct  41902  .....................-.............-.................................  41968
```

4E. Read_channel_234_read_0

```
Score               Expect   Identities         Gaps             Strand
3501 bits(3882)     0.0      2507/2765(91%)     173/2765(6%)     Plus/Plus

Query  61     TGTAGGCGAATT-GCGGAGCTATTGGGCCCT-TACAGGCATTCGGTACAGAGCGTGGCGTTAAGTGGTCAGACGAAGCGA--CTGGCTCTGGAGTGGAAAG---GATGGG-AGACAGGGCTGCAATGATAAATGTCGTTAGTTTCTCCGG  202
Sbjct  40958  ............T...........A.AG.T.A................................................GA.....................CGA.....G............-.........................  41106
                                                                                        <FLANKING 41053 TARGET>

Query  203    TGGCAGGACGTCAGCATATTTGCTCTGGCTAATGGGAGCAAAAGCGACGGGCAGGTAAAGACGTGCATTAC-TATTCATGGATAC--GATGTGAACATC-AATGACATATCGGTT-GTCAGGGAAGTTGTGAAGTTCTGGGATATAA-GC  346
Sbjct  41107  ...................................-...................................G.T...........AG.T..........C...............T..............................CC..  41255

Query  347    TCACCGTATTGCAGGTTGATA--AACCCGGAGCTTAGACAGCCTGACGTCGTATACGGTATGGGAACCAAAGGATATTCAGACGC--ATGCCTGTTCTGAAGCCATTTATCGATATGGTAAAGAAATATG-CACTCCATACGTCGGCGGC  491
Sbjct  41256  .....................TC............G.......AA.T.GT-..................................GA...........................................G...................  41404

Query  492    GC-TTCTTAATCGACATACGGAAACTCCGTTCCCTTCACCAAATACTGTGATGACCAGTTCGGGCG--GAATTTACACCACGTGGATTGGCATCAGAGCTGATGAACCGAGGCACCGGCTCAAAGCCAAAGCCTGGAATCAGATAT---G  635
Sbjct  41405  ..G....GC.CT....G.TTA.....-..............................T........AG.G.A......................................A..---....-.........................CTT.  41549

Query  636    CTGAACTGTGTCAGACTTTGAGAAGGAAGATATCCTCGCATGGTGGAAGCAACA-CGATTCGATTTGCAAATACCGGATCA-CTCGGGTGACTGCATATTCTGCATTAAAATC-CAACGCAAAA--TCGGAAT-GCCTGCAAAGATGAGG  779
Sbjct  41550  .........--...........................................A.C.....................A..T.....-.A.....................AAT..........AA.....C.T................  41696

Query  780    AGGGATTGCAGCGTGTTTT-AATGAGGTCATCACGG-ATCCCATGTGCGTGACGGACATCGGAGAACGCCAAAGGA--TTACTGTA-----GAAGAATGTCGCTCGACGGTATCGCCGAAAATGTATTCTCAGAAAATGATTATCAAGCC  920
Sbjct  41697  ...................T................G.........................GA............GA...-....CCGAG.............G............-...........--...................  41842

Query  921    CTGTACCCG-ACG-GGTACGAGCTAAAAGATTCGATACCGGCTCTTGTTCTGAGTCATGCGAAATATTTGGGAGGGCAGCTTGATTTCGACTTCGGGAGGGAAGCTGCATGATGCGATGTTATCGGTGCGGTGAATGCAAAGAAGATAAC  1068
Sbjct  41843  .....T.A.G..AT.........................................................-..............................................................................  41991

Query  1069   CGCTTCCGACCAAATCAACCTTACTGGAATCGATGGGTGTCTCC---GTGGAAGAACACCAACAGGGGGTGTTAC--CTACCGCAGG-AAAGGAGGACGTG----GCGACGGCGACGAAGTATCACCGACATAATCTGCG--AACTGCAA  1206
Sbjct  41992  ...........................-..................GGT...A...........-..........CA..........A.............TGGC.A...A.............................AA........  42139

Query  1207   ATACCTTCCAACGAAACGCACCAGAAAT-AACCCAAGCCAAT-CCAAAAGAATCTGACGT--AAACCTTCAACTACACGGCTCAC--TTGGGATAT-CGGTGG-TAAGAACGAAAGTCGTGCGAGGAAAACAAGGTGATTGACC-AAATC  1347
Sbjct  42140  ............................A.............C.................AA.......................CTG........C......C...-----.C..............................A.....  42284

Query  1348   GACAGTCATTGAACAAGAAAGCGTCGAGCGAGCTTTAACGTGCGCTAACTGCGGTCAGAAGCTGCATGTGCTGGAAGTTTTGACACGTGTGAG-GCACTGCTGCGCAGAACTCTGATGAGCGATCCGAATAGCTCCGATGCACGAGAGGA  1496
Sbjct  42285  ..-...T.-C.....................................................................----........T.A.................--.....................-........--.....  42423
                                                                                   <TARGET 42370 FLANKING>

Query  1497   AGAAGAGCTGATGGCTAAACCAGCGCGAAGACGATGTAAAAACGATGAATGCCTGGAATGGTTTCA---TGCATT--CTAATTGTATGGCGGTGCTCTCCGCAGAGTGT-GAACCAAGATAGCCACTCGAACGACGAAGGTAAAGAACGC  1640
Sbjct  42424  .....--..............................................G............CCC......CG...-.CAG...T...........--........G...........-.................-.........  42566

Query  1641   G-AAAAGCGGAAAAACAGCAGCAGAGAAGAAACGACGACGAGAGGAGCAGAAACAGAAAGATAAACTTAAGATTCGAAAACTCGC--TTAACTCCCGCAGTTACTGGATTGGACA--CTCAACAAGCCTCGTAAACGCCTTCATCAG-GA  1784
Sbjct  42567  .A............--.....................................................................CT.A..GC.................AA...AG.C.......--...................A..  42712

Query  1785   AAGAGACCGCGACTTACCATGTATCTCGTGCGGAACGCTCACGTCTGCTCAGTGGGATGCCGGACATTACCGGACAACTGCTGCGGCACCTCGACTCTGACGATTTAATGAAACGC-ATATTCACAAGCAATTGCGTG--GTGCAACCAG  1931
Sbjct  42713  ............................................................................................A...---...........-.....A..............-......GT..........  42857

Query  1932   CAC--AACCGGAAATCTCGTTCCGTATCGCGTCGAACTGATTAGCCGCATC-GGCAGGAAGCAGTAGACGAAATCGGTGAATCAAAACCCTATAGACGCCATCGCTGGACTATCGAAGAGTGC---GCAATCAATAAGCAGTACCAACAG  2075
Sbjct  42858  ...AA..G...........................................G.......................---.........---.C...AC..........................AAG..G.....GGCAG...........  43001

Query  2076   AAACTCAAAGACCTGCGAAATAGCAGAAGTGAGGCCGCATGACGTTC-CAGT-AAAACCATTCCAGACATGCTCGTTGAAACATACGGAAGTAAGAC-GAAGTAGCACGCAGACTGAAATGTAGTCGC-GTACGGTCAGAAAATACGTTG  2221
Sbjct  43002  ...............................................T....A...........................G.........A.C....A..............................G.....................  43151

Query  2222   ATGATAAAGACGGGAAAATGCACGCCATCGTCAACGACGTTCTCATGGTTCATCGCGGATGGAGTGAAAGAGATGCGCTATTAC-TAAAAATTGATGGCATCCAAATACCGAAATATTTGGTAGTAGTTGGCGATCTGCACGGATGCTAC  2370
Sbjct  43152  .....................................................................................GA.............-G...................--...........................  43298

Query  2371   GACGAACCTGATGAACAAACTGGATACGATTGGATTCAAC-----AAAAGTAATGCTTACCT--GTGGGCGATTTGGTTGATCGTGGTGCAGAGAACGTTGAATGCCT-GAATTAAT-ACATTCCCCTGGTTC-CAGCTGTACGTGGAAA  2510
Sbjct  43299  -....................................G..AACAA.....ACC......T..CG............................................G........C...............AG...............  43447

Query  2511   CCATGAGCAAATGATGATTGGTGGCTTATCAG-CCGTGGAAACGTTAATCCACTGGCTGCTTAAT-GCGGTGGCTGGTTCTTTAATCTC-ATTACGACAAAGAAATTCTGGCTAAAGCTCTTGCCCATAAAGCATACGAACTT-CGTTAA  2656
Sbjct  43448  ....................A...........AG...............-...............G........................G...........................................G.T......C......  43596

Query  2657   TCGGCTCGAACTGGTGAGCCAAAGATAA-----ACTTTATCTGCCACGCCGATTATCCCTTTGAC  2716
Sbjct  43597  ..--A..............-........AAAAT.TG.............................  43658
```

Supplementary Protocol Outline

Reagent list / provider

MinION gDNA Sequencing Kit MAP003	Oxford Nanopore Technologies
Platinum SYBR Green qPCR SuperMix-UDG	Invitrigen 11733-038
Platinum Taq DNA Polymerase	Invitrogen 10966-018
LongAmp Taq 2x Master Mix	NEB M0287S
QIAquick Gel Extraction Kit	Qiagen 28704
SeqCap Hybridization and Wash Kits	Roche 05634261001
Dynabeads M-270 Steptavidin	Invitrogen 65305
Agencourt AMPure XP PCR Purification beads	Beckman Coulter A63880
g-TUBE	Covaris 520079
Quant-iT PicoGreen dsDNA	Invitrogen P11496
Human Cot-1 DNA	Invitrogen 15279-011
NEBNext End Repair module	NEB E6050S
NEBNext dA tailing module	NEB E6053S
NEBNext Blunt/TA Ligase	NEB M0367S
Tris EDTA	Sigma
NaCl	Sigma

Procedure:

1. Preparation of biotin-adapters for baits

- Resuspend both complementary adapter oligonucleotides at the same molar concentration (10mM) using annealing buffer (10mM Tris 1mM EDTA pH 7.5-8.0, 50 mM NaCl)

- Merge equal volumes to make 5mM per oligo. Make 20ul per bait preparation.

- Incubate in thermal cycler at 95°C for 5 min and ramp down to 20°C with a rate of 1°C/min

- Keep on ice / -20. This is the **Biotin-adapter MIX.**

2. Baits generation and mix

- Run **2X** the following PCR reaction for each bait primer set (optimization is needed for each primer set / target)

94°C	2 min	10x buffer	5ul
		dNTPs	1ul
94°C	30 s	MgCl2	1.5ul
60°C	30 s	Platinum Taq	0.5ul
72°C	40 s	H2O	35ul
	35 cycles	Primer Forward	1ul
72°C	4 min	Primer Reverse	1ul
4°C	hold	Template DNA	5ul
		volume/reaction	50ul

- Load 2 wide wells per pcr-product in a 0.7-0.8% agarose gel
- Gel extract (2 bands per column) using QIAquick Gel Extraction Kit eluting in **30 ul** EB buffer
- Quantify with Picogreen
- Mix equimolar amounts of DNA adjusting to the lower bait yield
- Keep on ice / -20. This is the **Baits MIX.**

3. Genomic DNA/Baits treatment

- Shear the Genomic DNA at 5,000-7,000bp with Covaris g-tubes
- Dilute ~2-4ug of Baits MIX and 1-2ug of source DNA to 85ul final volume in TE.
- Perform End-Repair in parallel by adding:

  85ul sheared Genomic DNA + 10ul reaction buffer + 5ul enzyme mix

  85ul Baits Mix + 10ul reaction buffer + 5ul enzyme mix
- Incubate at 20°C for 30min
- Clean up the end-repaired Baits Mix with **1.8x** and the end-repaired Genomic DNA with **1x** Agencourt AMPure XP beads. Elute in 30ul and in 25ul TE respectively.
- Keep end-repaired Baits Mix on ice
- Perform dA-Tailing by adding:

  25ul end-repaired genomic DNA + 3ul buffer + 2ul enzyme
- Incubate at 37°C for 30min
- Ligate the adapters by adding:

  30ul end-repaired Baits Mix + 20ul Biotin adapters + 50ul 2xBlunt/TA Ligase mastermix

  30ul dA-tailed Genomic DNA + 20ul MinION PCR adapters + 50ul 2xBlunt/TA Ligase mastermix
- Incubate at RT for 20 min
- Clean up the biotin-adapter-ligated Baits Mix with **1.8x** and the PCR-adapter-ligated Genomic DNA with **1x** Agencourt AMPure XP beads. Elute both in 16ul TE.
- Quantify with Picogreen
- Keep adapter ligated products on ice / -20.

4. Hybridization and streptavidin beads capture

- Prepare the following hybridization mix:

PCR-adapter-ligated genomic DNA	15ul (~500ng)
*Biotin-adapter-ligated Baits Mix	**8ul (1000x target copies excess)
Human cot-1 DNA	5ul
2x Hybridization buffer (vial5)	(Final volume/2) ul
Hybridization component A (vial6)	(Finale volume/10) ul

*Add the baits after the initial denaturation step as described in *SeqCap Hybridization and Wash Kit* protocol.

**Dilute accordingly

- Follow the *SeqCap Hybridization and Wash Kit* protocol up to the final washing step of streptavidin beads.
- Resuspend the pelleted beads in 48ul PCR-grade water. This is the **PCR template** in the following reaction.
- Setup PCR reaction by adding:

50ul LongAmp Taq 2x mastermix + 2ul MinION primers (barcoded if necessary) + 48ul PCR-template

- Run the following program in a thermocycler:

95oC 3 min
95oC 15 s<br>62oC 15 s<br>65oC 4 min<br>18 cycles
65oC 8 min
4oC hold

5. Library finalization and MinION sequencing run

- Separate beads with a magnet and keep the amplified product
- Quantify with Picogreen. Expected mass of DNA is > 1ug
- Dilute 1ug of PCR product to 80ul of PCR-grade water. Add 5ul DNA CS.
- Follow MinION protocol for ligating the sequencing adapters and load the sequencer for a 48-hours run. Reload library every 12 hours.
